# Covalent Proteomimetic Inhibitor of the Bacterial FtsQB Divisome Complex

**DOI:** 10.1021/jacs.2c06304

**Published:** 2022-08-09

**Authors:** Felix M. Paulussen, Gina K. Schouten, Carolin Moertl, Jolanda Verheul, Irma Hoekstra, Gregory M. Koningstein, George H. Hutchins, Aslihan Alkir, Rosa A. Luirink, Daan P. Geerke, Peter van Ulsen, Tanneke den Blaauwen, Joen Luirink, Tom N. Grossmann

**Affiliations:** † Department of Chemistry and Pharmaceutical Sciences, Vrije Universiteit Amsterdam, De Boelelaan 1085, Amsterdam 1081 HV, Netherlands; ‡ Medical Microbiology and Infection Control (MMI), Amsterdam UMC Location VUmc, De Boelelaan 1108, Amsterdam 1081 HZ, Netherlands; § Amsterdam Institute of Molecular and Life Sciences (AIMMS), Vrije Universiteit Amsterdam, De Boelelaan 1085, Amsterdam 1081 HV, Netherlands; ∥ Department of Molecular Microbiology, Vrije Universiteit Amsterdam, De Boelelaan 1085, Amsterdam 1081 HV, Netherlands; ⊥ Department of Bacterial Cell Biology and Physiology, Swammerdam Institute for Life Sciences, University of Amsterdam, Sciencepark 904, Amsterdam 1098 XH, Netherlands

## Abstract

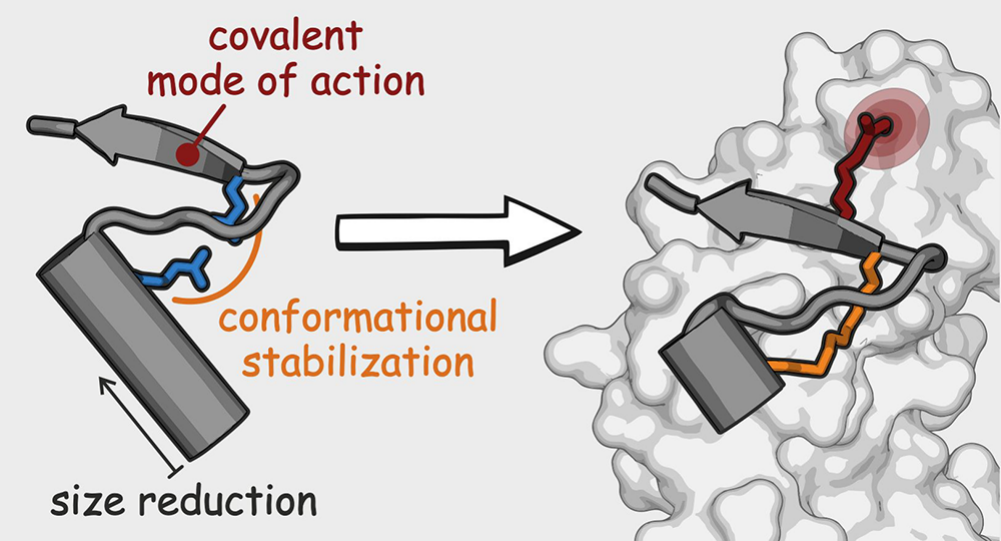

The use of antibiotics is threatened by the emergence and spread of multidrug-resistant strains of bacteria. Thus, there is a need to develop antibiotics that address new targets. In this respect, the bacterial divisome, a multi-protein complex central to cell division, represents a potentially attractive target. Of particular interest is the FtsQB subcomplex that plays a decisive role in divisome assembly and peptidoglycan biogenesis in *E. coli.* Here, we report the structure-based design of a macrocyclic covalent inhibitor derived from a periplasmic region of FtsB that mediates its binding to FtsQ. The bioactive conformation of this motif was stabilized by a customized cross-link resulting in a tertiary structure mimetic with increased affinity for FtsQ. To increase activity, a covalent handle was incorporated, providing an inhibitor that impedes the interaction between FtsQ and FtsB irreversibly*.* The covalent inhibitor reduced the growth of an outer membrane-permeable *E. coli* strain, concurrent with the expected loss of FtsB localization, and also affected the infection of zebrafish larvae by a clinical *E. coli* strain. This first-in-class inhibitor of a divisome protein–protein interaction highlights the potential of proteomimetic molecules as inhibitors of challenging targets. In particular, the covalent mode-of-action can serve as an inspiration for future antibiotics that target protein–protein interactions.

## Introduction

**Figure 1:**
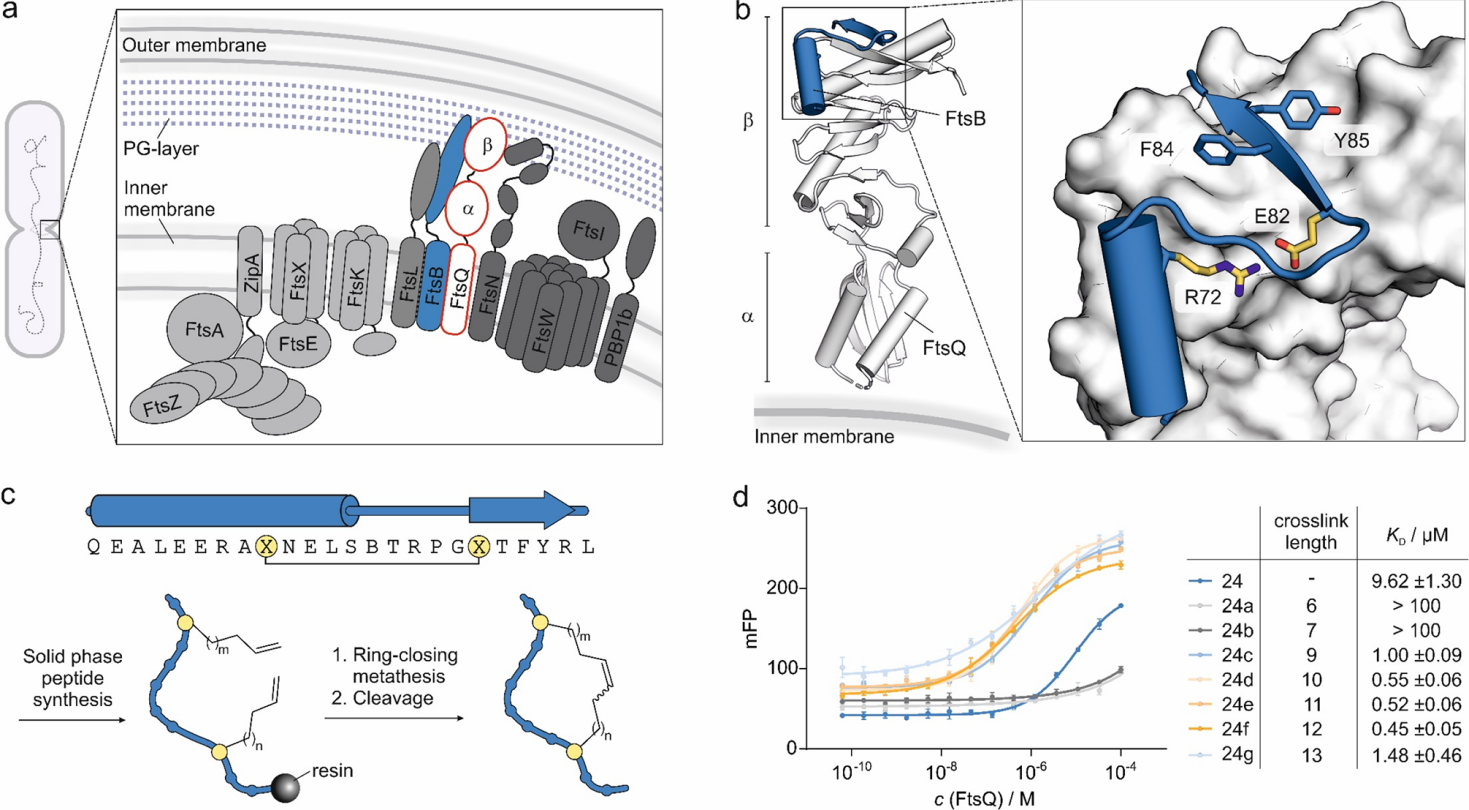
(a) Scheme of the divisome complex showing key subunits. Early assembly proteins (light gray) are recruited to the midcell plane first. FtsA and ZipA anchor FtsZ protofilaments on the inner membrane. FtsZ is a prokaryotic tubulin homologue and forms a ring at the nascent division site, the so-called Z ring, during cell division. This ring serves as a platform for the peptidoglycan synthesis machinery.^45^ FtsK is recruited to assist in chromosome segregation and in turn recruits the FtsQBL complex, which serves as a structural hub for recruitment of late assembly proteins (dark gray). Interaction of FtsN with the FtsQBL complex indicates the completion of divisome complex formation.^11,12^ (b) Crystal structure (PDB: 6h9o) of periplasmic FtsQ domains (white) in a complex with FtsB-derived peptide **24** (blue). FtsB residues essential for binding are shown in a stick representation.^23^ (c) Top: sequence of peptide **24**-derived macrocycles including associated secondary structure elements in the FtsQ-bound state. Bottom: synthesis sketch of macrocyclic peptides. (d) Fluorescence polarization (FP) measurements using fluorescein-labeled analogues of **24** (*c* = 10 nM) and FtsQ(50–276) (*c* = 0.06 nM–100 μM). The table provides *K*_D_-values for macrocyclic peptides bound to FtsQ(50–276). Peptide cross-link lengths are indicated (6–13 carbon atoms; for peptide details, see Table S1). All measurements were performed in triplicate (*n* = 3 replicates, error bars = SD).

The discovery of antibiotics represents one of the main advances in the history of medicine. However, in recent years, this achievement is threatened by the emergence and spread of multidrug-resistant bacterial strains.^1^ A WHO study revealed that the situation is critical for healthcare-associated infections caused by Gram-negative species such as certain strains of *Escherichia coli* (*E. coli*) that belong to the ESKAPE group of highly virulent pathogens.^2^ Moreover, newly developed antibiotics often affect already established targets and therefore suffer analogous drawbacks.^3,4^ Thus, there is a need for antibiotics that act via novel modes of action and address new targets.^5−9^ Here, the inhibition of bacterial cell division has moved into the focus of antibiotic research. Central to cell division is the divisome,^10,11^ a dynamic complex composed of numerous membrane-associated proteins that assemble at the midcell plane to regulate cell constriction, peptidoglycan synthesis, and cell separation. Divisome assembly involves sequential and precisely orchestrated protein–protein interactions (PPI) with imbalances ultimately leading to cell death.^12^ In Gram-negative bacteria, efforts to target the divisome have focused on the inhibition of FtsZ polymerization in the cytoplasm, which represents one of the initial steps of bacterial cell division (Figure 1a), however, without providing potent inhibitors.^13−17^ Consequently, the validation of alternative divisome targets is needed to further explore the potential of divisome inhibition in Gram-negative bacteria.

One such target is the FtsQB divisome subcomplex that plays a central role in cell division connecting early and late recruitment steps during divisome assembly (Figure 1a).^18−22^ The interaction of FtsQ with FtsB occurs mainly in the periplasm,^23,24^ which is located between the inner and outer membranes of Gram-negative bacteria. Since it is enclosed by only one membrane, the periplasm is more accessible to inhibitors than the bacterial cytoplasm. Other advantages of FtsQ targeting include its low cellular abundance (20 to 300 copies per cell)^25,26^ and its conservation among Gram-negative bacteria.^26^ Furthermore, while there is a human homologue of FtsZ, this is not the case for FtsQ, which potentially allows for more selective targeting.^27,28^ A crystal structure of the complex between periplasmic domains of *E. coli* FtsQ and FtsB^23^ reveals a 24-amino acid FtsB sequence interacting with the β domain of FtsQ (Figure 1b). The importance of this interface has also been confirmed by site-directed photocross-linking and mutagenesis studies.^21,26^ These findings highlight the relevance of the FtsQB complex for cell division and suggest that the inhibition of this PPI offers antibiotic potential.

Previous efforts to target the FtsQB interaction with small molecular scaffolds have failed to provide inhibitors,^29^ which is in line with the general challenges associated with PPI inhibition. This can be explained by the large and shallow interaction areas of most PPI and the frequent lack of well-defined binding pockets.^30^ Thus, small molecular scaffolds used in classic drug discovery often fail to provide potent and selective PPI inhibitors. As an alternative, peptide-based scaffolds have been pursued utilizing the unique surface-recognition properties of proteins.^30−32^ Notably, the mimicry of small tertiary folds has proven to be effective for particularly challenging targets.^33−40^ These so-called proteomimetics encompass multiple secondary structure elements^41^ and provide high-affinity binders when single secondary-structure motifs have failed.^35−38^ Importantly, structure-based design strategies can provide straightforward access to proteomimetic PPI inhibitors given the availability of a structurally characterized protein complex.^30,42^ However, due to their relatively large molecular weight, proteomimetic inhibitors tend to exhibit low cellular uptake,^43,44^ which complicates their use for intracellular targets, in particular, for Gram-negative bacteria.

Here, we report the structure-based design of an FtsB-derived proteomimetic in which a key intramolecular salt bridge was replaced by a hydrocarbon bridge. The initially obtained 24-mer macrocyclic peptide showed high affinity for FtsQ (*K*_D_ = 0.5 μM) but low antibiotic activity. Subsequent shortening of the peptide sequence and installation of a covalent modifier provided an antibiotic agent capable of inhibiting a membrane-permeable *E. coli* strain*.* Most importantly, this activity was concurrent with the expected loss of FtsB localization. Finally, the covalent inhibitor also affected growth of a clinical *E. coli* strain in a zebrafish larvae infection model.

## Results and Discussion

### FtsB-Derived Peptides Bind to FtsQ

The crystal structure of a FtsQB complex^23^ shows the 24-mer FtsB sequence adopting a small tertiary motif that involves an N-terminal α helix and a C-terminal β-strand, which are connected by a seven-amino acid turn structure (FtsB amino acids 75–82, Figure 1b). An intramolecular salt bridge between R72 and E82 links the two terminal secondary structures (α-helix and β-strand) thereby stabilizing the S-shaped tertiary motif. Notably, this salt bridge is highly conserved among bacterial FtsB homologues^26^ and was found to be essential for cell division in mutagenesis studies.^23^ To stabilize the S-shaped conformation of the FtsB motif and thereby promote FtsQ binding, the salt bridge was replaced by a covalent hydrocarbon cross-link. This type of cross-link has previously been used on isolated α-helices^30,46^ but not in the context of tertiary motif stabilization. A library of FtsB-derived peptides with a varying number of bridging atoms was generated by the introduction of different combinations of non-natural olefin-bearing amino acids at the bridging positions (X, Figure 1c). Subsequently, peptide macrocyclization was performed using ring-closing metathesis (Figure 1c).^47−49^ Initially, we observed low cyclization yields, which were improved by the introduction of a pseudoproline building block (at positions L75–S76; Figure S1) during the solid-phase peptide synthesis. The building block is located between the two cross-linking sites presumably bringing those in closer proximity.^50^ To facilitate affinity measurements using a fluorescence polarization (FP) readout, peptides were N-terminally modified with fluorescein isothiocyanate (FITC), which was attached via a polyethylene glycol (PEG_2_) linker (Figure S1). Consistent with previous reports, the linear precursor **24** exhibited moderate affinity (*K*_D_ = 9.6 ± 1.3 μM; Figure 1d). Depending on the cross-link length, the peptide affinity for FtsQ varied considerably. While short cross-links (6 and 7 carbon atoms) resulted in the complete loss of binding, longer cross-links increased the affinity for FtsQ. This observation is in line with the expected distance between the peptide backbones at the two cross-linking sites in the bound state (Figure S2). The highest affinity derivative is macrocyclic peptide **24f** (*K*_D_ = 0.45 ± 0.05 μM), which harbors a 12-carbon cross-link and shows a 21-fold increased affinity when compared to linear peptide **24**.

Next, macrocycle **24f** was examined regarding its activity on *E. coli* growth. It is important to note that the interface of the FtsQB complex is localized in the periplasm, which requires a potential inhibitor to cross the outer membrane (OM). However, the OM exhibits low permeability to molecules larger than ∼600 g/mol,^43,44^ which potentially hampers the uptake of **24f** (MW = 2913 g/mol). To improve the periplasmic uptake and test the general feasibility of the targeting approach, the *E. coli* mutant *lptD*4213 (imp) was used. This strain exhibits a defect in the transport of lipopolysaccharides, which results in decreased OM integrity and thus facilitates the access of medium-sized structures into the periplasm.^51,52^ For this reason, *E. colilptD*4213 (imp) has already been used in antibiotic discovery efforts.^51^ To assess bacterial growth, the optical cell density (OD_600_) was monitored upon treatment with N-terminally acetylated versions of peptide **24** and **24f**. However, we did not observe inhibitory activity for these peptides (*c*_max_ = 150 μM Figure S3).

### Covalent Modifier Facilitates Antibiotic Activity

Potential reasons for the lack of **24f**-induced growth inhibition include its insufficient affinity for FtsQ and/or uptake into the periplasm. Both would prevent efficient inhibition of FtsQB complex formation.^19,20^ A strategy to increase the apparent target affinity utilizes the formation of a covalent linkage between the inhibitor and protein of interest. This can be achieved by installation of a reactive group (modifier) that addresses a particular amino acid on the target protein.^53−58^ Inhibitor binding brings the modifier and the target amino acid in proximity, which results in high local reactant concentrations and dramatically accelerated reaction rates.^56^ The covalent linkage of the ligand and target then prevents dissociation and results in an extremely prolonged residence time. Such covalent inhibitors have been applied for a number of challenging targets.^57−59^ Inspired by these examples, we aimed to convert macrocycle **24f** into a covalent inhibitor.

**Figure 2:**
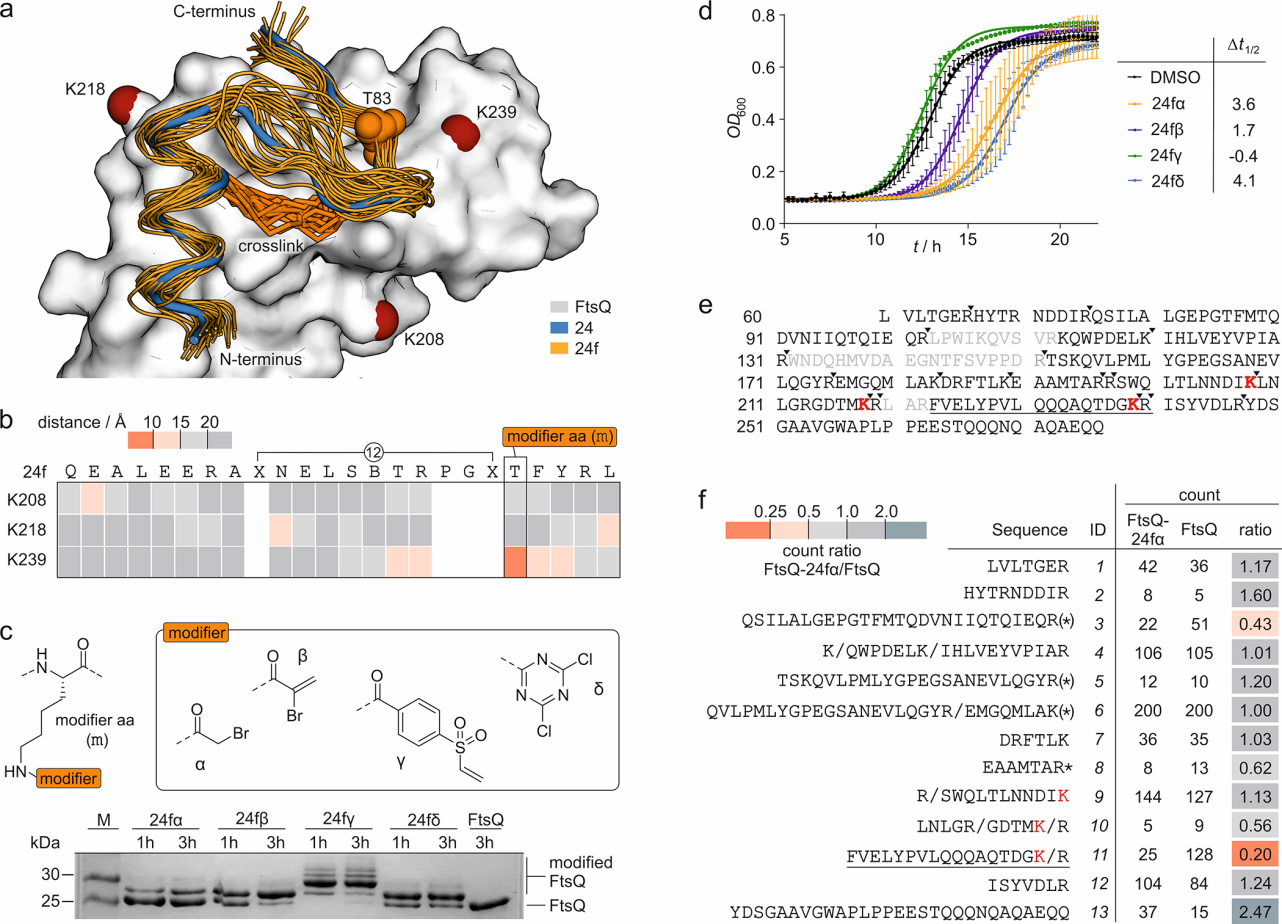
(a) Overlay of peptide **24** (blue, crystal structure, PDB: 6h9o) bound to FtsQ (white) with binding poses of **24f** (orange) derived from MD simulations (snapshots every 10 ns from three independent 100 ns simulations; the pdb file with atomic coordinates is provided in the Supporting Information). The cross-links in **24f** (orange stick representation) and FtsQ lysine residues in proximity to the binding site (*d̅* < 15 Å, red spheres for Nε) are highlighted. (b) Heat map of the average distance between the Nε of the selected lysine and the Cβ of **24f** residues over a 400 ns MD simulation (Table S2). (c) Top: structure of the four selected modifiers (α, β, γ, and δ) installed in a modified amino acid (m), which was introduced at position T83 of **24f**. Bottom: 17% Tris/Tricine PAGE (protein modification assay) assessing peptide binding to FtsQ(50–276). Covalent inhibitors **24fα**–**δ** (*c* = 125 μM; for peptide details, see Table S3) were incubated with FtsQ(50–276) (*c* = 50 μM) for 1 or 3 h. Up-shifted bands are indicative of modified FtsQ. (d) Growth assay using *E. colilptD*4213 (imp) after treatment with inhibitors **24fα**–**δ** (*c* = 50 μM). Optical density at 600 nm (*OD*_600_) was measured every 15 min over 22 h. Measurements were conducted in triplicate (*n* = 3 technical replicates, error bars = SD). (e) Sequence coverage for unmodified FtsQ(50–276) after tryptic digest in the MS/MS experiment (only sequence fragments with a count of >1 were included, and missing sequence fragments are shown in gray; the solid inverted triangle indicates the protease cleavage site). (f) List of identified sequences (count of >1). Counts for **24fα**-modified and unmodified FtsQ as well as the corresponding count ratio are shown (* oxidized methionine, (*) oxidized and non-oxidized methionine, / additional cleavage site; for full list of fragments, see Table S4).

To support the design of **24f**-based covalent inhibitors, we initially assessed the binding mode of **24f** by molecular dynamics (MD) simulations. For that purpose, a model of **24f** bound to FtsQ was generated using the structure of the **24**/FtsQ complex (PDB: 6h9o) as template. MD simulations were performed with Amber20 (see Methods in the Supporting Information for details).^60^ In brief, FtsQ and the peptide were parameterized using the ff14SB force field, while parameters for the hydrocarbon bridge were defined with the general Amber force field (GAFF).^60^ Initially, three independent 100 ns MD simulations were performed (Figure S4) and snapshots from 10 ns intervals were analyzed (orange, Figure 2a). The MD-derived binding poses of **24f** (orange) sample a space around the bound conformation of linear **24** (blue) in a complex with FtsQ in the previously reported crystal structure (Figure 2a).^23^ Analogous to the salt bridge in **24**, the hydrocarbon cross-link forms an interface with the FtsQ binding site. In addition, a 400 ns MD simulation was performed, confirming that the binding conformation of **24f** remains stable throughout this longer trajectory (Figure S4).

Covalent inhibitors usually employ electrophilic modifiers that target nucleophilic residues on the protein, primarily cysteine, histidine, or lysine.^57,58^ Using our **24f**/FtsQ complex model, we screened the vicinity of the binding site (*d̅* < 15 Å) for these residues. While FtsQ does not harbor a cysteine or histidine near the **24f** binding site, there are three lysine residues (K208, K218, and K239). To identify potential sites for the introduction of the modifier, average distances between the Nε of each lysine and the Cβ of each **24f** residue were determined based on the 400 ns MD simulation. In this analysis, the bridging amino acids as well as proline and glycine were excluded due to their expected importance for peptide conformation. This analysis (Figure 2b) reveals FtsQ residue K239 and **24f** residue T83 as the only pair with an average distance of less than 10 Å (*d̅* = 7.5 Å, Figures S5 and S6). Consequently, amino acid position T83 in **24f** was selected for the introduction of a covalent modifier.

We considered the testing of 11 different electrophiles^61−65^ (α–φ, Figure S8) to identify the most suitable candidates. To reduce the synthetic effort in this initial screening round, linear peptide **24** was used as the ligand. Instead of T83, an orthogonally protected lysine (Figure S7, Fmoc-K(MMT)-OH, MMT: mono-methoxy trityl) was introduced during the solid-phase peptide synthesis of **24**. Mildly acidic conditions allowed the selective cleavage of MMT followed by the installation of the corresponding electrophile via amide formation or nucleophilic aromatic substitution. The resulting library of modified peptides (Table S3) was then incubated with FtsQ(50–276) and protein modification-assessed by Tris/Tricine PAGE. Here, modified proteins appeared as an up-shifted band (Figure S8). This initial screen revealed that the four modifiers 2-bromoacetamide (α), 2-bromoacrylamide (β), 4-(vinylsulfonyl)benzamide (γ), and 4,6-dichloro-1,3,5-triazin-2-amine (δ) provided clearly up-shifted bands after 3 h of incubation (Figure S8). Subsequently, these four modifiers (α, β, γ, and δ, Figure 2c) were implemented in macrocyclic peptide **24f**. The obtained macrocyclic modified peptides (**24fα**, **24fβ**, **24fγ**, and **24fδ**) again showed labeling of FtsQ(50–276). However, the vinylsulfon-modified peptide **24fγ** caused multiple up-shifted bands that indicate non-specific reactions with multiple protein residues. With this panel of potential covalent inhibitors in hand, bacterial growth assays were performed by employing the permeable *E. colilptD*4213 (imp) strain (Figure 2d). Here, both **24fα** and **24fδ** showed a considerable delay in growth (Δ*t*_1/2_ = 3.6 and 4.1 h, respectively) when compared to the DMSO-treated sample.

Before pursuing further inhibitor optimization, we were interested if the covalent modifier indeed targets FtsQ K239. For that purpose, unmodified as well as **24fα**-modified FtsQ(50–276) were subjected to tryptic digest and subsequently analyzed using HPLC-coupled high-resolution tandem mass spectrometry (MS).^66^ For unmodified FtsQ(50–276), identified fragments covered amino acids 60–276 with three intervening stretches missing (sequences: 103–112, 131–151, and 219–222, Figure 2e). Importantly, the three lysine residues (K208, K218, and K239, Figure 2a) in proximity to the binding site of **24f** are within the covered regions (Figure 2f), and they are located in different peptide fragments (K208: ID-9, K218: ID-10, and K239: ID-11). After treatment with **24fα** and tryptic digest, an analogous sequence coverage was obtained. When comparing the abundance (counts) of fragments of **24fα**-modified and unmodified FtsQ(50–276), most sequences show changes within a twofold margin (0.5–2.0 count ratio; Figure 2f). Notably, only two sequences experience more than twofold reduced abundance in the **24fα**-modified version (ID-3 and ID-11, count ratio = 0.43 and 0.20, respectively). Among those, only the more severely reduced sequence ID-11 (fivefold reduction) contains a lysine, namely, the anticipated target residue K239. This suggests a covalent modification of lysine K239. We were, however, not able to detect a corresponding modified peptide fragment when searching for different possible modifications as well as alternative truncation patterns.^66^ This may be due to low solubility or poor ionization behavior of the resulting covalently modified peptide fragment. Importantly, sequences ID-9 and ID-10, containing lysines K208 and K218, do not show severe count reductions for **24fα**-modified FtsQ (count ratio = 1.13 and 0.56, respectively). Taken together, these results support the anticipated residue K239 as the most likely site of covalent modification.

### Inhibitor Truncation Increases Antibiotic Activity

**Figure 3:**
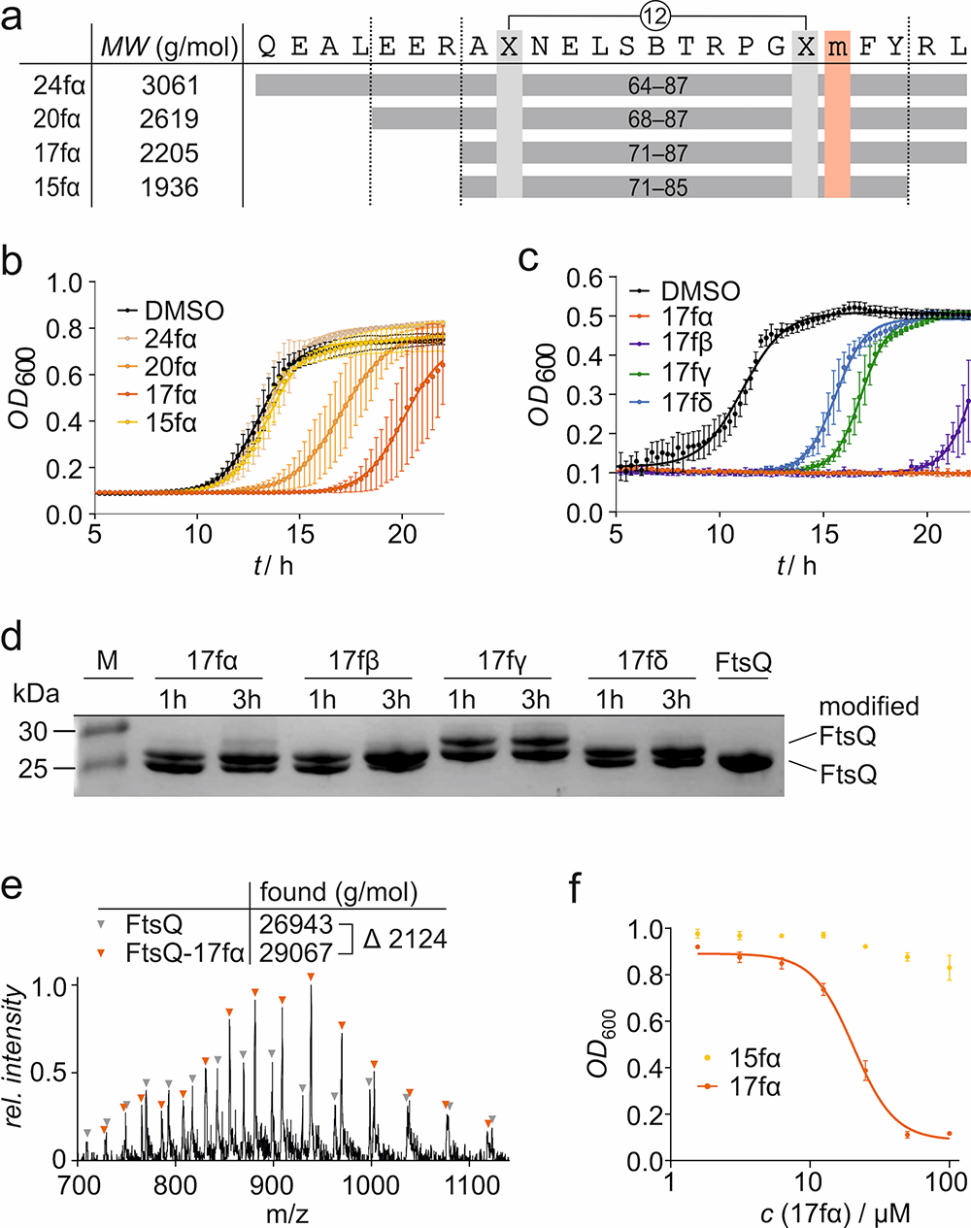
(a) Overview of truncated versions of **24fα**. The positions of the cross-linking amino acids (X, light gray) and of the modifier-bearing amino acid (m, red) are indicated (for peptide details, see Table S3). (b) Growth assay using *E. colilptD*4213 (imp) after treatment with **24fα**, **20fα**, **17fα**, and **15fα** (*c* = 25 μM). The optical density at 600 nm (OD_600_) was measured every 15 min over 22 h. Measurements were conducted in triplicate (*n* = 3 technical replicates, error bars = SD). (**c**) Growth assay using *E. colilptD*4213 (imp) after treatment with **17fα**–**δ** (*c* = 50 μM). The optical density at 600 nm (OD_600_) was measured every 15 min over 22 h. Measurements were conducted in triplicate (*n* = 3 technical replicates, error bars = SD). (d) 17% Tris/Tricine PAGE (protein modification assay) assessing peptide binding to FtsQ(50–276). Covalent inhibitors **17fα**–**δ** (*c* = 125 μM; for peptide details, see Table S3) were incubated with FtsQ(50–276) (*c* = 50 μM) for 1 or 3 h. Up-shifted bands are indicative of modified FtsQ. (e) MS of FtsQ(50–276) after **17fα** treatment. Signals corresponding to unmodified (gray) and **17fα**-labeled FtsQ(50–276) are indicated. Obtained masses (obtained by deconvolution) are shown (calculated masses, FtsQ: 26939 g/mol, FtsQ-17fα: 29061 g/mol). The obtained mass difference (ΔMW = 2124 g/mol) corresponds well with the one expected after a reaction with 1 equiv of **17fα** (ΔMW = 2122 g/mol; for details, see Figure S9). (f) Concentration-dependent effect of **17fα** and **15fα** (*c* = 0.78 μM–2100 μM) on the growth of *E. colilptD*4213 (imp) after 15 h. All measurements were performed in triplicate (*n* = 3 replicates, error bars = SD).

Knowing that the uptake into the periplasm depends on the molecular weight of the inhibitor, we tested how truncations of the peptide sequence affect antibiotic activity. For that purpose, three truncated versions of bromoacetamide-modified **24fα** were designed (Figure 3a). Bromoacetamide was initially chosen due to its straightforward synthetic implementation. Two peptides were truncated at the N-terminus, lacking either four (**20fα**) or seven amino acids (**17fα**), as well as one peptide on both sides, lacking seven amino acids at the N-terminus and two at the C-terminus (**15fα**). To study the effect of inhibitor truncation, bacterial growth assays employing the permeable *E. colilptD*4213 (imp) strain were performed (*c*(inhibitor) = 25 μM). These experiments revealed increased inhibitor activity upon N-terminal shortening (activity: **17fα** > **20fα** > **24fα**; Figure 3b). However, C-terminally truncated version **15fα** did not exhibit inhibitory activity.

As covalent inhibitor **17fα** showed the highest inhibitory activity, the 17-mer scaffold was next tested with the remaining three modifiers (β, γ, and δ, Figure 3c) at increased peptide concentrations (*c* = 50 μM) to enable clear discrimination between the different inhibitors. While all compounds affected bacterial growth, vinyl sulfone-modified **17fγ** and dichlorotriazin-modified **17fδ** showed the smallest effects (Δ*t*_1/2_ = 4.5 and 5.7 h, respectively). The highest activity is observed for bromoacetamide-modified **17fα**, which prevents bacterial growth under these conditions (Figure 3c). To verify covalent inhibition of FtsQ(50–276), the four covalent inhibitors were examined in the PAGE-based protein modification assay (Figure 3d). Here, all inhibitors show efficient protein labeling; however, modification with vinyl sulfone-bearing **17fγ** resulted in two up-shifted bands that indicate multiple modifications. Taken together, bromoacetamide-modified inhibitor **17fα** shows the highest inhibitory activity as well as robust and selective covalent modification.

To investigate **17fα** activity in more detail, we confirmed the covalent modification of FtsQ using HPLC/MS. After **17fα** treatment of FtsQ(50–276), MS spectra revealed the occurrence of a protein species with an increased molecular weight (ΔMW = 2124 g/mol; Figure 3e), which was in line with the mass difference upon a reaction with **17fα** (ΔMW = 2122 g/mol; Figure S9). In addition, the concentration-dependent effect of **17fα** on the growth of *E. colilptD*4213 (imp) was explored, revealing an inhibitory effect at concentrations as low as 12.5 μM (Figure 3f). Notably, peptide **15fα**, lacking two C-terminal amino acids when compared to **17fα**, did not result in growth inhibition (highest tested concentration, *c* = 100 μM).

### Inhibitor Affects the Bacterial Phenotype

**Figure 4:**
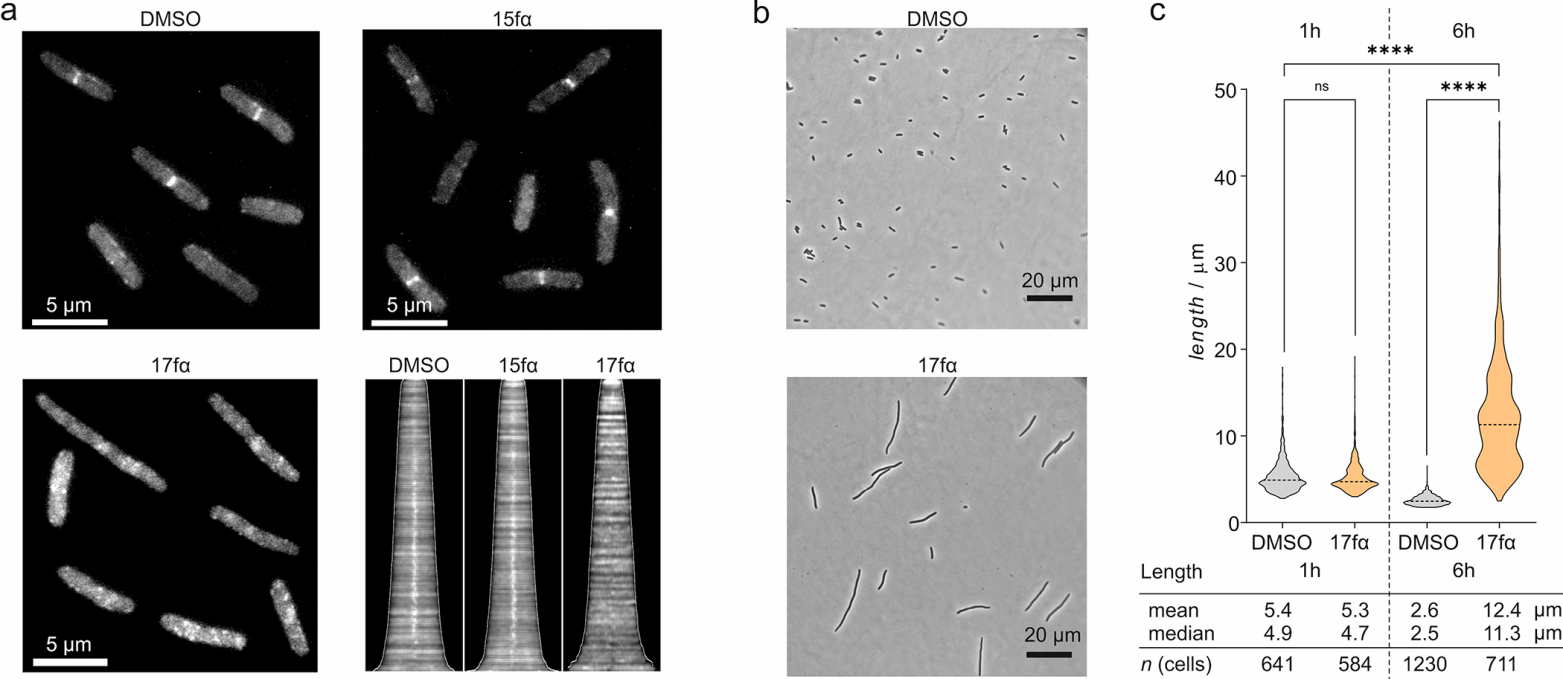
(a) Representative fluorescent microscopy pictures (scale bar = 5 μm) showing FtsB-mNG localization (white) in *E. colilptD*4213 (imp) in the presence of either DMSO, **15fα**, or inhibitory peptide **17fα** (both *c* = 100 μM) 60 min after addition. The corresponding demograph shows the fluorescence intensity along the longitudinal axis of treated cells (cells ordered by lengths). The midcell fluorescence intensity consistent with the presence of FtsB-mNG is lost for **17fα**-treated cells as indicated by the absence of a white signal at the center of the *x* axis compared to the others. (b) Morphology of representative *E. coliLptD*4213 cells treated with either DMSO or **17fα** (*c* = 100 μM) after 6 h (scale bar = 20 μm). The violin plot shows the corresponding cell length distribution of samples treated with either DMSO or 100 μM **17fα** after 1 and 6 h. Significance was determined by the Kruskal–Wallis test and Dunn’s multiple-comparison test (ns: *p* > 0.05, ****p < 0.0001).

To further examine the effect of **17fα** on *E. colilptD*4213 (imp), we investigated FtsB localization, which was expected to change upon interference with the FtsQB interaction. During bacterial cell division, FtsQ recruits FtsB to the midcell plane.^67^ Hence, inhibition of the FtsQB interaction should result in a loss of FtsB accumulation at the midcell. To visualize FtsB, *E. colilptD*4213 (imp) was transformed with a plasmid encoding FtsB fused to the fluorescent reporter protein NeonGreen (FtsB-mNG) under the control of a weak p*tcr*99A promoter.^68^ The cells were grown in the presence of **17fα**, **15fα**, or DMSO only. After induction of FtsB-NG expression, phase contrast and fluorescence microscopy pictures were taken (Figure 4a). After 1 h of incubation, DMSO and **15fα** treatment showed the regular accumulation of FtsB-mNG at the midcell of dividing cells. Consistent with the inhibition of the FtsQB interaction, incubation with **17fα** indeed led to the loss of midcell localization with individual cells showing an elongated character (Figure 4a). Notably, the effect on FtsB localization was concentration and time-dependent (Figure S10).

Mutational studies have shown that interference with the FtsQB complex formation can result in cell elongation and filamentation of *E. coli* cells.^23^ To investigate the effects of **17fα** on bacterial morphology, *E. colilptD*4213 (imp) cells were incubated with **17fα** (*c* = 100 μM) for a prolonged period (up to *t* = 6 h). Analysis by phase contrast microscopy and subsequent quantification using ImageJ with an ObjectJ Cell Counter plugin^69^ revealed a time-dependent increase in the average cell lengths upon incubation with **17fα** (Figure 4b,c and Figure S11). DMSO treatment on the other hand slightly reduced the cell lengths, which is consistent with an expanding bacterial population and the associated depletion of nutrients causing bacteria to enter the stationary growth phase.^70^ Taken together, the observed delocalization of FtsB (Figure 4a) and increased cell lengths upon treatment with covalent inhibitor **17fα** point toward interference with the divisome function and support a mode-of-action that involves targeting of the FtsQB complex.

### Antibiotic Activity in Zebrafish

So far, cell-based assays were performed with *E. coli* mutant *lptD*4213 (imp) possessing a permeable OM thereby supporting periplasmic uptake of the inhibitors. To assess the activity of **17fα** in a more relevant context, the clinical multidrug-resistant *E. coli* 87 strain was chosen. In a corresponding growth assay, high inhibitor concentrations (*c* > 100 μM; Figure S12) were required for inhibition to indicate indeed reduced periplasmic uptake in this strain. We have recently reported the membrane-active peptide **L8S1** that can increase the uptake of large scaffold antibiotics across the outer membrane.^71^ To test the impact of **L8S1** on **17fα** activity, the fractional inhibitory concentration index (FIC_index_; Figure S12) was determined based on the minimum inhibitory concentrations (MIC) of **17fα**, **L8S1**, or their combinations in a checkerboard synergy assay.^72^ In fact, **L8S1** potentiates the activity of **17fα** (FIC_index_ = 0.33), indicating improved periplasmic uptake.

**Figure 5:**
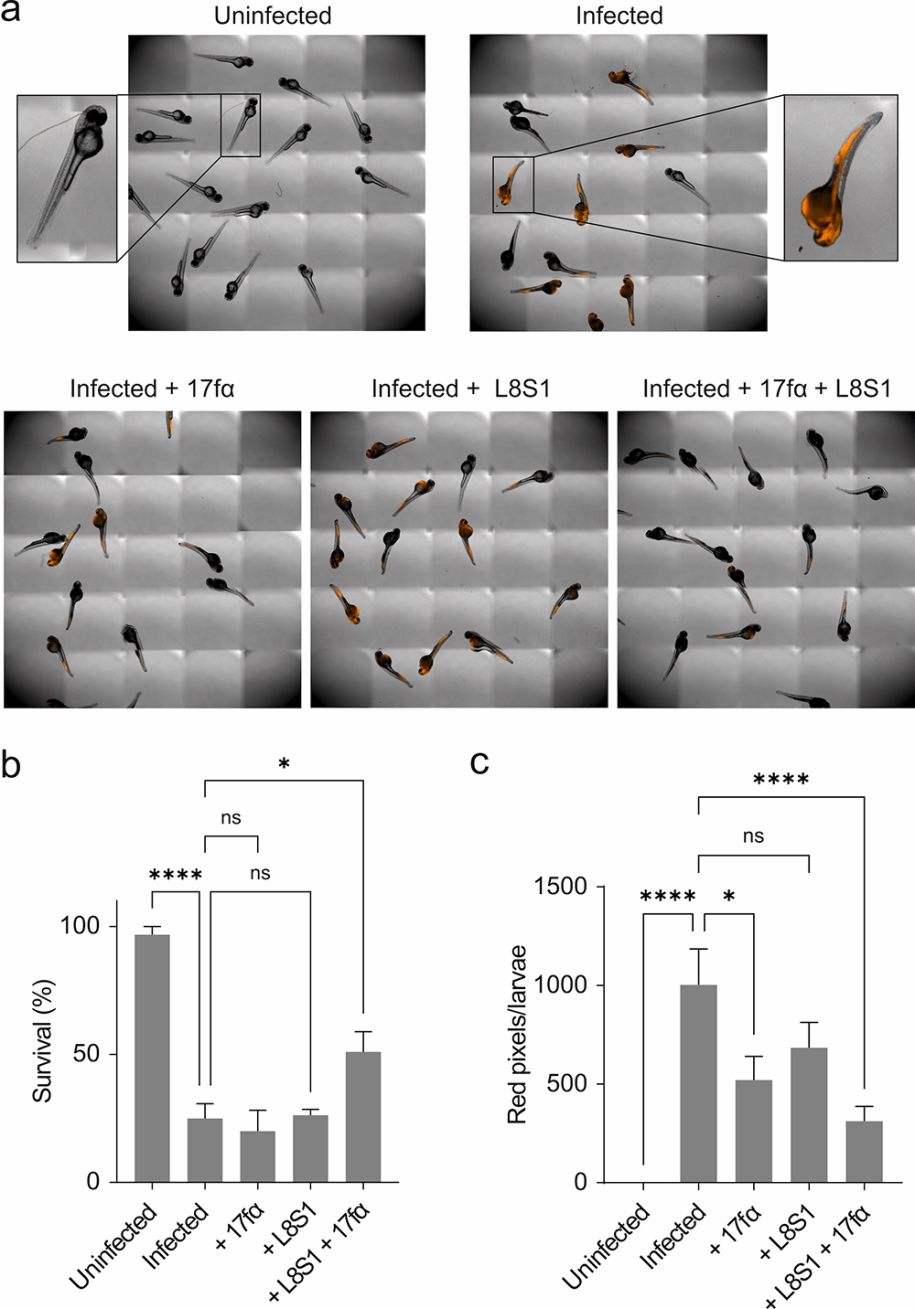
(a) Representative pictures of uninfected and infected zebrafish larvae. Top left: Uninfected larvae. In the remaining pictures, zebrafish larvae were infected with *E. coli* 87 (CFU = 255) encoding fluorescent mScarlet. Subsequently, they were either treated with **17fα** (*c* = 70 μM), **L8S1** (*c* = 3.125 μM), or a combination of both. (b) Relative share of surviving zebrafish larvae after 24 h as determined by heartbeat depending on the treatment regime. (c) Effect of different treatments on the infection progress after 24 h as determined by quantification of red pixels per larvae (zebrafish larvae pictures can be found in Figure S14). All measurements were performed in quadruplicate (*n* = 4 replicates with 15 Zebrafish larvae per condition, error bars = SEM). Significance was determined by one-way ANOVA and Dunnet’s multiple-comparison test (*p < 0.05, **p < 0.01, ***p < 0.001, and ****p < 0.0001).

The observed synergy between **L8S1** and **17fα** prompted our interest in testing the combination of both agents in vivo. We selected a zebrafish model involving transparent Casper zebrafish (*Danio rerio*) larvae, which were infected with *E. coli* 87. In brief, zebrafish larvae were individually microinjected with *E. coli* 87 that had been transformed with a plasmid encoding the fluorescent protein mScarlet.^73−75^ This allowed monitoring of the infection progress by fluorescence microscopy (Figure 5a). After infection with *E. coli* 87, zebrafish larvae show a clear fluorescent signal (right) in contrast to the uninfected population (left, Figure 5a). In addition, survival of the larvae was assessed based on heartbeat showing a 72% reduction in larvae survival upon *E. coli* 87 infection (Figure S13). To assess antibiotic activity of the inhibitor, zebrafish larvae were microinjected with solutions of **17fα** and **L8S1**, individually or in combination. Initially, the general compound toxicity was assessed by treating uninfected larvae, which did not reveal signs of toxicity at tested compound concentrations (Table S6). Infected zebrafish larvae were then treated with **17fα**, **L8S1**, or a combination (1 h post *E. coli* 87 infection). Notably, combined treatment with **17fα** and **L8S1** (*c* = 70 and 3.125 μM, respectively) resulted in a higher survival rate when compared to individual treatment or non-treated larvae (Figure 5b). The combination of both agents also resulted in a decreased bacterial load as indicated by a significantly reduced signal based on fluorescence microscopy (bottom; Figure 5a). Individual compound treatments showed a less pronounced reduction in the bacterial load (Figure 5c).

## Conclusions

Bacterial cell division is an intricate process that is carefully orchestrated via the dynamic formation of the divisome, a complex of membrane-associated proteins at the midcell. Interference with divisome complex formation can lead to cell division arrest, filamentation, and eventually cell death. Hence, the divisome represents an appealing antibiotic target. Here, we addressed the essential interaction between divisome proteins FtsQ and FtsB^26^ in a structure-based approach aiming at the interaction interface of their periplasmic domains. An FtsB-derived peptide (residues 64–87) served as the starting point comprising an essential intramolecular salt bridge (between R72 and E82),^23^ which we replaced with a covalent cross-link. This resulted in proteomimetic ligand **24f** with a 21-fold increase in affinity when compared to the linear starting sequence. Proteomimetic **24f** represents a rare case in which macrocyclization was utilized to stabilize a mini tertiary fold^34−36,40^ and is distinct from the typical use of so-called staples to stabilize α-helical secondary structures by bridging neighboring turns within an isolated helix.

Macrocyclization alone did not result in potent antibiotic activity, prompting us to pursue the development of a covalent inhibitor to increase activity. MD simulations suggested FtsQ lysine K239 as a potentially suitable modification site. Additionally, K239 has been described as a highly conserved residue among 246 γ-proteobacterial homologues of *E. coli*, supporting a crucial role in FtsQ function,^26^ which can be expected to reduce the occurrence of resistant mutants.^76^ Different modifiers were tested regarding their ability to selectively target FtsQ. Among the tested electrophiles, moderately reactive bromoacetamide proved to be the most suitable, showing selective modification of FtsQ. Subsequently, the peptide sequence was truncated to support periplasmic uptake. The resulting 17-mer covalent inhibitor **17fα** showed the highest antimicrobial activity among the tested compounds presumably due to a favorable combination of affinity, reactivity, and uptake characteristics.

Initial tests have been performed with *E. coli* mutant *lptD*4213 (imp) possessing a permeable outer membrane. Here, **17fα** was found to affect cell length and FtsB localization, pointing toward the FtsQB interaction as the target. The inhibitor also increased survival and delayed the infection progress in a zebrafish larvae model using the clinical multidrug-resistant *E. coli* 87 strain. This effect on an *E. coli* strain with regular membranes is notable and might be explained by an excess of lysozyme in zebrafish, resulting in a more permeable outer membrane.^77^ Covalent inhibitor **17fα** is the first compound to inhibit the FtsQB interaction and highlights the divisome as a potential drug target. In this respect, **17fα** represents an appealing starting point for the future development of more effective, potentially smaller FtsQB interaction inhibitors. Notably, the targeting strategy can be expected to be transferable to other Gram-negative bacteria due to the high conservation of the FtsQB complex. Finally, the presented approach is an uncommon example of a peptide-based covalent inhibitor targeting a non-catalytic amino acid.^78−85^ This highlights the potential of proteomimetic molecules with a covalent mode-of-action as inhibitors of challenging protein–protein interactions.

## Supporting Information Available

Detailed methods: peptide synthesis and purification, implementation of modifiers, protein expression and purification, fluorescence polarization assay for affinity measurements, molecular dynamics (MD) simulations, protein modification assay, LC/MS-analysis of modified FtsQ(50-276), growth assays, microscopy and morphology assay, localization of FtsB-mNG, plasmid construction, and microinjection of zebrafish larvae with *E. coli* 87; supporting figures and tables, and peptide structures and analytics (PDF)
Coordinates of MD-derived structures (PDB)

## Supplementary Material

ja2c06304_si_001.pdf

ja2c06304_si_002.pdb
