# Supplementary material for: Covalent Proteomimetic Inhibitor of the Bacterial FtsQB Divisome Complex
Source: J Am Chem Soc. 2022 Aug 9;144(33):15303–13. doi: 10.1021/jacs.2c06304 (PMC9413201; doi:10.1021/jacs.2c06304)
Supplement: Supplementary file 1 — ja2c06304_si_001.pdf [file ja2c06304_si_001.pdf]

Supporting information

Covalent proteomimetic inhibitor of the bacterial FtsQB divisome complex

Felix M. Paulussen[1,2,3], Gina K. Schouten[4], Carolin Moertl[1,2], Jolanda Verheul[5], Irma Hoekstra[1], Gregory M. Koningstein[2,3], George H. Hutchins[1,2], Aslihan Alkir[1], Rosa A. Luirink[1,2], Daan P. Geerke[1,2], Peter van Ulsen[2,3], Tanneke den Blaauwen[5], Joen Luirink[2,3,*], Tom N. Grossmann[1,2,*]

[1] Department of Chemistry and Pharmaceutical Sciences, Vrije Universiteit Amsterdam, De Boelelaan 1085, 1081 HV Amsterdam, The Netherlands

[2] Amsterdam Institute of Molecular and Life Sciences (AIMMS), Vrije Universiteit Amsterdam, De Boelelaan 1085, 1081 HV Amsterdam, The Netherlands

[3] Department of Molecular Microbiology, Vrije Universiteit Amsterdam, De Boelelaan 1085, 1081 HV Amsterdam, The Netherlands

[4] Medical Microbiology and Infection Control (MMI), Amsterdam UMC Location VUmc, De Boelelaan 1108,1081 HZ Amsterdam, The Netherlands

[5] Department of Bacterial Cell Biology and Physiology, Swammerdam Institute for Life Sciences, University of Amsterdam, Sciencepark 904, 1098 XH Amsterdam, The Netherlands

* Correspondence: t.n.grossmann@vu.nl (T.N.G.); s.luirink@vu.nl (J.L.)

Table of contents

1. Supporting methods 3
1.1 Peptide synthesis and purification 3
1.1.1 Olefin crosslink 4
1.1.2 N-terminal modification 4
1.1.3 Implementation of modifiers 4
1.2 Protein expression and purification 5
1.2.1 Transformation and expression 5
1.2.2 Isolation and purification 5
1.3 Fluorescence polarization assay for affinity measurements 5
1.4 Molecular Dynamics (MD) parameterization and simulations 6
1.5 Protein modification assay 6
1.6 LC/MS-analysis of modified FtsQ(50-276) 7
1.7 Growth assays 7
1.8 Microscopy and morphology assay 7
1.9 MS/MS experiment 8
1.10 Localization of FtsB-mNG in the presence of FtsQ-FtsB inhibitory peptide 9
1.11 Checkerboard synergy assay 10
1.12 Plasmid construction 10
1.13 Microinjection of zebrafish larvae with *E.coli* 87 expressing mScarlet 11
2. Supporting tables 12
3. Supporting figures 18
4. Peptide structures and analytics 33
5. References 53

1. Supporting methods

1.1 Peptide synthesis and purification

General

Peptides were generated using Fmoc-based solid-phase synthesis as described in protocols earlier.[1–3] Reagents were used without any additional purification and were purchased from Iris Biotech GmbH (Marktredwitz, Germany), Sigma Aldrich (Darmstadt, Germany), Carl Roth (Karlsruhe, Germany) and Okeanos Biotech (Beijing, China). Peptide sequences were assembled using an automated peptide synthesizer (Syro I, MultiSynTech GmbH, Witten, Germany). Peptides were synthesized on H-Rink amide ChemMatrix® resin (Sigma-Aldrich, Art. No. 727768, Darmstadt, Germany). Unless stated otherwise, all procedures were performed with 1 mL of solvent or reagent solution per 50 mg resin for all scales (10–100 µmol). The resin was swollen in DMF (dimethylformamide) for 30 min prior to usage.

Amino acid coupling

A double coupling protocol of 4 eq. PyBOP (1st 40 min coupling, benzotriazol-1-yl-oxytripyrrolidino-phosphonium hexafluorophosphate) and 4 eq. HATU (2nd 40 min coupling, hexafluorophosphate azabenzo-triazole tetramethyl uranium) as coupling reagents and DMF as solvent was used. Additionally, the coupling reaction with 4 eq. Fmoc-protected amino acid was supplemented with 4 eq. Oxyma and 8 eq. DIPEA. A capping step was performed using Ac_2O and DIPEA in NMP(1:1:8, v/v/v) for 10 min (2x). Fmoc removal was conducted using 25% (v/v) piperidine in DMF (2x).

After every double coupling, a capping step was performed using Ac_2O and DIPEA in DMF (1:1:8) for 10 min. In case of a PG sequence motif, N-alpha-(9-Fluorenylmethyloxycarbonyl)-L-prolinyl-glycin (Iris-Biotech GmbH) was used. In case of a LS sequence motif, a pseudo-proline ((S)-3-(N-(9-Fluorenylmethyloxycarbonyl)-L-leucinyl)-2,2-dimethyloxazolidine-4-carboxylic acid, Iris Biotech GmbH) was used.

Peptide cleavage and purification

Cleavage of peptides from resin and removal of side chain protecting groups was performed simultaneously by incubating the resin with a solution of 94% trifluoroacetic acid (TFA), 2.5% H_2O and 1% triisopropylsilane (TIPS) twice for 1.5 h. The combined cleavage solution was subsequently reduced under nitrogen flow. Peptides were precipitated using Et_2O at -20 °C and subsequently centrifuged at 4000 rpm for 10 min. The supernatant was decanted and the precipitated peptide lyophilized. Cleaved and lyophilized peptides were dissolved in a solution of 20% acetonitrile (ACN) in water (+ 0.1% TFA) and purified by reversed-phase HPLC on an Agilent semi preparative system 1100 (Macherey-Nagel Nucleodur C18 column; 10×125 mm, 110 Å, 5 µm particle size) using a flow rate of 6 $mL \cdot min^{-1}$ and various gradients of solvent A (H_2O + 0.1% TFA) and solvent B (ACN + 0.1% TFA) over 20-40 min. Obtained pure fractions were pooled and lyophilized.

Characterization

Characterization of peptides was performed by analytical reverse-phase HPLC (1260 Infinity, Agilent Technology; flow rate of 1 mL·min^{-1}, A: water with 0.1% FA and 0.01% TFA, B: ACN with 0.1% FA and 0.01% TFA; Agilent Eclipse XDB-C18 column, 4.6×150 mm, 5 µm particle size) using a 30 min gradient (5-95% B) coupled to a mass spectrometer (6120 Quadrupole LC/MS, Agilent Technology, Santa Clara, CA, United States) using electrospray ionization. Analytical HPLC chromatograms at 210 nm and MS spectra, masses and *m*/*z*-values are shown in the appendix (analytics part). Quantification of acetylated peptides was performed with a NanoDrop OneC using calculated extinction coefficients (https://pepcalc.com/; accessed on 1 February 2018; ε_W = 5690 M^{-1}cm^{-1}, ε_Y = 1280 M^{-1}cm^{-1}, ε_C = 120 M^{-1}cm^{-1}) for λ = 280 nm.

1.1.1 Olefin crosslink

For macrocyclization of olefinic non-natural amino acids, ring closing metathesis was performed.[4] Fmoc-protected non-natural olefinic amino acids (Okeanos Tech, Beijing, China) were incorporated in peptide synthesis and treated as natural amino acids. After synthesis, the resin with immobilized peptide was washed and swollen in dichloroethane (DCE) for 15 min. A solution of 4 mg·mL^{-1} benzylidene-bis(tricyclohexylphosphine)dichloro-ruthenium (Grubbs Catalyst™ 1st generation) in DCE was added to the resin and reacted at room temperature (RT) for 1.5 h. This procedure was repeated until a sufficient quantity of crosslinked peptide was observed in analytical LC/MS. After metathesis, the resin was washed with DCE, dichloromethane (DCM) and DMF three times, respectively.

1.1.2 N-terminal modification

The final Fmoc deprotected peptides were subsequently FITC-PEG_2 labelled for affinity measurements or acetylated for cell-based experiments. N-terminal acetylation was performed twice with Ac_2O and DIPEA in DMF (1:1:8) for 10 min. Prior to FITC labelling, the flexible spacer 8-(9-fluorenylmethyloxycarbonyl-amino)-3,6-dioxaoctanoic acid (PEG_2) was coupled like an Fmoc-protected amino acid and deprotected as stated above. Fluorescein-isothiocyanate (FITC) was implemented using 4 eq. FITC isomer I and 8 eq. DIPEA in DMF for 1.5 h twice.

1.1.3 Implementation of modifiers

For modifier implementation, an Fmoc-protected lysine with a monomethoxytrityl (MMT) protected sidechain (N-alpha-(9-Fluorenylmethyloxycarbonyl)-N-epsilon-4-methoxytrityl-L-lysine) was incorporated during peptide synthesis. After metathesis and N-acetylation of the N-terminus, the MMT protected lysine sidechain was selectively deprotected using a solution of 1 % TFA and 2 % TIPS in DCM until absence of yellow coloring (appr. 5x 30 min). The resin was washed 5 times with DCM and then DMF. Modifiers bearing a carboxylic acid handle (4 eq.) were implemented using 4 eq. of Oxyma (ethyl cyano(hydroxyimino)acetate), 4 eq. of COMU ((1-cyano-2-ethoxy-2-oxoethylidenaminooxy)dimethylamino-morpholino-carbenium hexafluorophosphate) and 4 eq. of DIPEA in DMF for 20 min. Modifier δ (2,4,6-trichloro-1,3,5-triazine) (4 eq.) was coupled using 8 eq. of DIPEA in THF at 0 °C for 1h. The procedures were repeated (maximum of three repetitions) until a high quantity of the desired product was observed in analytical LC/MS.

1.2 Protein expression and purification

Plasmids were obtained from Luirink et al. as described in previous publications.[5]

1.2.1 Transformation and expression

Escherichia coli BL21 DE3 were freshly transformed with pET16b FtsQ(50-276), a His_6 fusion construct of the periplasmic domain of FtsQ in a modified pET16b vector. After heat shock transformation, bacteria were grown on an agar plate with 100 µg·mL^{-1} ampicillin as selection marker at 37 °C overnight. A single colony was used to inoculate 100 mL lysogeny broth (LB) medium (1 g tryptone, 0.5 g yeast extract, 1 g NaCl, add 100 mL ddH_2O, pH 7.4) with 100 µg·mL^{-1} ampicillin. Cells were grown at 37 °C and 200 rpm overnight. 50 mL of this overnight pre-culture were used to inoculate 1 L LB medium (100 µg·mL^{-1} ampicillin) and incubated at 37 °C and 200 rpm. After the culture reached an optical density (OD_{600}) of 0.8 (λ = 600 nm), expression was induced with 1 mM isopropyl ß-D-1-thiogalactopyranoside (IPTG) and the cells were grown until an OD_{600} of 1.5 was reached. After harvesting the cells by centrifugation at 4000 rcf and 4 °C for 15 min, the cell pellet was washed in 6 mL phosphate-buffered saline solution (137 mM NaCl, 2.7 mM KCl, 10 mM Na_2HPO_4, 2 mM KH_2PO_4). The suspension was centrifuged, the supernatant was discarded and the washed pellet was shock frozen in liquid N_2 and stored at -80 °C until further use. For protein purification, the pellet was resuspended in buffer I (50 mM sodium phosphate pH 7.5, 500 mM NaCl, 4 % glycerol and 10 mM imidazole) to a total volume of 100 mL, before DNase, lysozyme and 100 µM PMSF were added. The mixture was incubated on ice for 5 min. The cells were then disrupted using the Microfluidizer 1109 (15000 PSI). After four cycles of homogenization, the cell debris was removed by centrifugation at 70.000 rcf at 10 °C for 45 min.

1.2.2 Isolation and purification

To isolate FtsQ(50-276), the soluble fraction was applied to a 5 mL HiTrap TALON crude prepacked column (Cytivia) and purification performed on an AKTA FPLC system (Cytivia). After a wash with 2 column volumes (CV) buffer I the His_6-fusion protein was eluted with high imidazole buffer II (50 mM sodium phosphate pH 7.5, 500 mM NaCl, 4 % glycerol, 400 mM imidazole) using a stepwise gradient (Gradient: 1 CV 5% buffer II, 5 CV 24.3% buffer II, 2 CV 100% buffer II; 2 mL fractions). In a final step, the buffer was exchanged and the protein concentrated in buffer III (10 mM HEPES, pH 7.4, 150 mM NaCl and 4 % glycerol) using Amicon Ultra Centrifugal Filters (10 kDa cut off). After concentration FtsQ was frozen in liquid nitrogen and stored at -80°C in 10 mM HEPES, pH 7.4, 100 mM NaCl, 4 % glycerol. Fresh aliquots were used in every experiment.[2]

1.3 Fluorescence polarization assay for affinity measurements

To determine the affinity of the peptides their FITC-labelled versions were dissolved in assay buffer (25 mM HEPES, pH 7.4, 100 mM NaCl, 0.01 % Tween-20) to provide a 40 nM peptide solution. A 3-fold dilution of FtsQ(50-276) protein (15 µL per well) was presented in a 384-well plate (Corning, black, flat bottom) and incubated with the peptide solution (5 µL, final peptide concentration c = 10 nM), starting with highest protein concentrations ranging from 0.02 µM to 100 µM. After incubation at room temperature for 1 h, fluorescence polarization was measured using a Tecan Spark 20M plate reader with λ_{ex} = 485 nm and λ_{em} = 525 nm. K_D -values were determined by nonlinear regression analysis of dose-response curves using GraphPad Prism software.[2]

1.4 Molecular Dynamics (MD) parameterization and simulations

A structure of the FtsB-bound 24f peptide was designed using Avogadro software[6] utilizing the crystal structure of the FtsQ-FtsB complex as a template (PDB: 6h9o). The Cβ atom of R72 was connected to the Cβ of E82 by the cross-linker (residue name STP) containing a total of 10 additional carbon atoms, including a trans double bound between the central 5th and 6th carbon atoms. The geometry of the staple was optimized, avoiding clashes either internally or with the bound FtsB molecule. The arginine and glutamate residues involved in bonding to the staple were re-defined as modified alanine residues (name AST) for parameterization.

GAFF-based (general AMBER force field) parameters were obtained to describe the staple structure. Initially, partial charges were derived using antechamber (Amber tools[7]) using the AM1-BCC charge model, calculated for a methyl-capped structure of the staple. Complete parameters for the 10-carbon staple were generated using the prepgen application, with additional parameters added manually to describe the bonding between the staple and connecting side chains. Amber parameters for the non-canonical norleucine residue within the peptide were available and applied from the Forcefield_NCAA[8] library. All relevant parameters, input files, and structural snapshots are included in the following Github depository (https://github.com/georgehutch/FtsQ_staple_MD).

Multiple independent MD simulations of FtsB in complex with both wild-type FtsQ and stapled 24f were calculated, including three 100 ns and one 400 ns simulations of the FtsB/24f complex to ensure thorough sampling of potential binding poses. Protonation of the complex was determined using the H++ server[9] and input files were generated using tleap including solvation and neutralization in TIP3P water box, followed by system minimization, heating and NPT equilibration. Production MD simulations were executed with the Amber20 pmemd.cuda application using the Bazis HPC cluster (VU Amsterdam) and trajectories analyzed using cpptraj.

1.5 Protein modification assay

To determine the reactivity of the different modifiers FtsQ(50-276) was diluted to a concentration of 100 µM in 20 mM NH_4HCO_3 buffer (pH 7.6, 150 mM NaCl). The individual peptides were diluted to a concentration of 250 µM in the same buffer. Equal volumes of both solutions were mixed and incubated at 37 °C for the given time period (1 h or 3 h). The reaction was quenched using 5x SDS sample buffer (pH 6.8*,* 1.875 g TRIS-Cl, 25 mL glycerol, 5 g SDS, 12.5 mL β-mercaptoethanol, 0.5 % bromphenol blue and add 50 mL ddH$_2$O). Samples were heated to 96 °C for 2 min and subsequently analysed by 17 % tris/tricine PAGE (15 min at 90 V and then 3:45 h at 150 V). PageRuler™ Prestained Protein Ladder, 10 to 180 kDa (Thermo Scientific™) was used as a ladder. Staining was performed using standard Coomassie Staining Solution (40 mL EtOH, 0.3 g Coomassie Blue R250, 0.3 g Coomassie Blue G250, ad 100 mL ddH$_2$O.) and then destained using water.

17 % Tris/Tricine gel (2x):

Running gel: 1.6 mL H_2O, 5.5 mL AB-mix, 2.5 mL Buffer A, 100 µL 10% APS, 10 µL 1% TEMED

Stacking gel: 2.1 mL H_2O, 0.5 mL AB-mix, 0.38 mL Buffer A, 50 µL 10% APS, 5 µL 1% TEMED

Buffers:

Buffer A (4x) pH 8.45 3M Tris, 0.3 % SDS; Anode Buffer (10x) pH 8.8, 3M Tris; Cathode buffer (10x) 1M Tris, 1 M Tricine, 1 % SDS.

1.6 LC/MS-analysis of modified FtsQ(50-276)

To determine the reactivity of 17fα, FtsQ(50-276) was diluted to a concentration of 100 μM in 20 mM NH_4HCO_3 buffer (pH 7.6, 150 mM NaCl). The peptide 17fα was diluted to a concentration of 250 μM in the same buffer. Equal volumes of both solutions were mixed and incubated at 37 °C for the given time period (1 h or 3 h). The reaction was quenched by 4-fold dilution in ACN/H_2O (1:1) + 0.1 % TFA.

20 μL of the resulting solution were injected and analysed by analytical reverse-phase HPLC (1260 Infinity II, Agilent Technology; flow rate of 0.3 mL·min^{-1}, A: water with 0.1% FA, B: 80% iPrOH, 10% ACN, 10% H_2O with 0.1% FA; AdvanceBio RP-mAB C4 column, 2.1×50 mm, 3.5 μm particle size) using a 10 min gradient (20-80% B) coupled to a mass spectrometer (Infinity Lab LC/MSD XT, Agilent Technology) using API-ES (MSD signal settings: Polarity: Positive; scan mass range: 500-3000; Fragmentor 70). Analytical HPLC chromatograms at 210 nm and MS spectra, masses and *m*/*z* ratios are shown in the appendix – analytics part.

1.7 Growth assays

Growth assays were performed to assess the *in vitro* cytotoxicity of peptides. The experiments were executed under sterile conditions. For each assay, 20 mL of fresh MOPS medium was prepared (Teknova Inc MOPS EZ Rich Defined Medium Kit | M2105: 40 mM MOPS, 4 mM Tricine, 0.01 mM $FeSO_4$, 9.5 mM NH_4Cl, 0.276 mM K_2SO_4, 0.5 μM $CaCl_2$, 50 mM NaCl, traces of $(NH_4)_6Mo_7O_{24}$, H_3BO_3, $CoCl_2$, $CuSO_4$, $MnCl_2$ and $ZnSO_4$, 1.32 mM Dibasic Anhydrous KH_2PO_4, 0.20% Glucose, 1.5 mM KOH, 0.199 mM Adenine, Cytosine, Uracil and Guanine and 0.01 mM to 10 mM amino acids). A preculture of *E.coli lptD*4213 (imp) was prepared in 5 mL MOPS medium with Streptomycin (30 μg/mL) and incubated overnight at 200 rpm and 37°C. The next day, the overnight culture was diluted a thousandfold and grown to an OD_{600} of about 0.5 at 200 rpm and 37°C. 10 mM peptide stocks in DMSO were diluted to four times the desired highest concentration to be measured in MOPS medium. The peptide dilutions were vortexed and briefly centrifuged. 50 μL of MOPS medium was added to each well of a Corning® 96-well Clear Flat Bottom Polystyrene TC-treated Microplate. A twofold dilution series was performed by adding 50 μL of the diluted peptide to the top row, mixing it and taking 50 μL to the next row. At each step the solution was resuspended five times. This was repeated until the last row which was skipped in order to obtain a reference measurement. After 2-3 h, the *E.coli lptD*4213 (imp) pre-culture was diluted to an OD_{600} of 0.00005. Then, 50 μL of diluted culture were added to each well and the plate sealed using a lid and parafilm. The OD_{600} was measured every 15 min over a period of more than 20 h (continuously shaking, 37°C) using a BioTek Synergy H1 plate reader. The obtained data were analyzed and relative growth curves plotted with GraphPad Prism 8. The assays were performed as technical triplicates. A single overnight pre-culture was used to prepare the diluted MOPS culture used in the growth assay. The peptides of interest were diluted to the desired concentrations separately for each triplicate measurement. ^

1.8 Microscopy and morphology assay

Morphology assays were executed to assess the physiological change of *E.coli* cells upon exposure to different peptides. All steps in this protocol were performed under sterile conditions. For each assay, 20 mL of fresh MOPS medium was prepared (40 mM MOPS, 4 mM tricine, 0.01 mM $FeSO^{-4}$, 9.5 mM NH_4Cl, 0.276 mM K_2SO_4, 0.5

μM $CaCl_2$, 50 mM NaCl, traces of $(NH_4)_6Mo_7O_{24}$, H_3BO_3, $CoCl_2$, $CuSO_4$, $MnCl_2$ and $ZnSO_4$, 1.32 mM dibasic anhydrous KH_2PO_4, 0.20% glucose, 1.5 mM KOH, 0.199 mM adenine, cytosine, uracil and guanine and 0.01 mM to 10 mM amino acids). A preculture of *E.coli lptD*4213 (imp) was prepared in 5 mL MOPS medium with streptomycin (30 μg/mL), which was grown overnight at 200 rpm and 37°C. The next day, the overnight culture was diluted a thousandfold and grown up to an OD_{600} of about 0.5 at 200 rpm and 37°C. After 2-3h, the culture was diluted to an OD_{600} of 0.1. 10 mM peptide stocks in DMSO were subsequently diluted to 200 μM in MOPS medium. For measuring two time points, a total volume of 110 μL peptide dilution was prepared, for 3 timepoints this was 160 μL. For each time point, 50 μL of diluted peptide was added into an Eppendorf tube, to which 50 μL diluted culture was added (final peptide concentration: 100 μM, starting OD_{600} = 0.05). The tubes were incubated for either 1,3 or 6 h at 200 rpm and 37°C. Then the cells were fixed using 8.3 μL 37% formaldehyde solution in H_2O and incubated for 5 min at 200 rpm and 37°C. Subsequently, the mixture was centrifuged at 500 rpm for 5 min, after which the supernatant was discarded. The pellets were redissolved in 7.5 μL PBS buffer and stored at -20°C. [2]

Microscopy slides were prepared by melting 1% Nobel Agarose in MiliQ water in a microwave. 800 μL was dispersed over a well slide, a microscopy slide was put on top and air bubbles were removed. The agar was allowed to cool for 15 min, after which the microscopy slide was removed. The frozen samples were thawed and 3 μL of each sample was added to separate wells, after which a covering glass was put on top. Using an Olympus IX83 microscope with 100 times magnification ocular and phase contrast (halogen lamp wat approximately 7V), the morphology of the cells was investigated. Pictures were taken and subsequently analyzed with ImageJ and the ObjectJ Cell Counter (https://sils.fnwi.uva.nl/bcb/objectj/examples/CellCounter/cellcounter-md/cellcounter.html, accessed on February 2020).

1.9 MS/MS experiment

For the MS/MS experiment samples were prepared as follows. The FtsQ(50-276) stock solution was diluted to 100 μM and the stock of peptide 24fα was diluted to 250 μM with previously used binding buffer (20 mM NH_4HCO_3 (pH 7.6), 150 mM NaCl). 10 μL of each diluted stock were merged and the resulting mixture incubated over night at 37 °C. Then the samples were mixed with 5x SDS Sample buffer and 2.5 μL/lane loaded on to a 17% tris/tricine PAGE. The gel was run for 15 min at 90 V and 2h at 150 V.

A container of glass was prepared and extensively washed with soda and water. The gel was placed in the container and incubated in fixing solution (50% EtOH, 3% H_3PO_4) overnight to remove SDS. The next day, the fixing solution was removed and the gel washed with water a couple of times. Colloidal Coomassie staining solution (34% MeOH, 3 % H_3PO_4, 150 g $(NH_4)_2SO_4$/L, 1 g/L G250) was added and the gel stained for 2 d. Then the gel was washed 3x for 30 min with water. Then the bands corresponding to modified and unmodified FtsQ were cut out (1 mm^3 pieces) from the gel and transferred to original Eppendorf tubes. The gel pieces were washed with 200 μL water by vortex mixing for 30 s. Then the water was discarded. The gel pieces were then washed with 200 μL MeOH:50 mM NH_4HCO_3 (1:1 v/v) for 1 min with intermittent vortex mixing. The supernatant was discarded and the procedure repeated once. Then the gel pieces were dehydrated for 5 min in 200 μL of ACN: 50 mM NH_4HCO_3 (1:1 v/v) with intermittent vortex mixing. The supernatants were discarded and 200 μL of 100 % ACN added, mixed and incubated for 30 s. The samples were dried in a SpeedVac (appr. 5 min). They were then

rehydrated in 20 µL of 12 ng/µL Trypsin in 0.01% ProteaseMAX surfactant (Promega): 50 mM NH_4HCO_3 (Trypsin gold stock 1 µg/µl from Promega, high thermostability) for 10 min (2 µL to 200 µL) and overlayed with 30 µL 0.01 % ProteaseMAXsurfactant: 50 mM NH_4HCO_3 and gently mixed for several seconds. Then the samples were incubated for 1 h at 50 °C. The condensates were collected by centrifuging for 10 s. The digests were mixed for a few seconds and transferred to another tube. 0.5 % TFA was added to inactivate trypsin. The samples were dried in a SpeedVac at 30 °C for 15 min and stored at -20 °C.

Peptides were analyzed by LC-MS/MS using OrbiTrap and the results evaluated using Mascot Distiller (Matrix Science, London, UK) for data processing as described previously.[10–12]

1.10 Localization of FtsB-mNG in the presence of FtsQ-FtsB inhibitory peptide with *E.coli* mutant *lptD*4213 (imp)

Strain: *LptD*4213 *lptD4213*, *galK2*(Oc), λ^-, *IN(rrnD-rrnE)1*, *rpsL200*(strR), *rph-1*.[13] Plasmid: pNM105 pSAV057-FtsB-mNG^{EC} Cm^R, p15A origin of replication, protein is expressed under control of the $P_{trcdown}$ promoter.[14] Media: TY: 10 g Tryptone (Bacto laboratories, Australia), 5 g yeast extract (Duchefa, Amsterdam, The Netherlands) and 5 g NaCl (Merck, Kenilworth, NJ) per liter) supplemented with 0.5% glucose (Merck); MOPS: MOPS EZ Rich Defined Medium Kit M2105 (Teknova).

*LptD*4213 was transformed with pNM105 and stored in TY medium 15% glycerol at -80 °C. For each experiment, cells were taken from the -80 °C storage and grown during the day in TY at 37°C. At the end of the day, the cells were diluted in rich MOPS medium and grown overnight at 37 °C. The next day, the cells were grown and diluted to OD_{450} of 0.080 before adding them to wells in a microplate reader (Multiskan FC, Thermo Scientific). After 50 min when exponential growth was observed, the cells were diluted 1:1 by addition of the inhibitor. DMSO did not affect the growth rate of the strain up to 1%. For some wells growth was allowed to continue while other wells were used to take samples for microscopy after 0, 5, 15, 30 and 60 min. The cells were fixed for 15 min with 2.8% formaldehyde (Sigma-Aldrich) and 0.04% glutaraldehyde (Merck KGaA,) after being taken out of the plate reader wells. The samples were washed twice in PBS (140 mM NaCl, 27 mM KCl, 10 mM $Na_2HPO_4{\cdot}2H_2O$, 2 mM KH_2PO pH 7.2) and allowed to mature overnight at RT, washed once more with 1 ml PBS and were then imaged.

For imaging the cells were immobilized on 1% agarose in PBS slabs on object glasses as described[15] and photographed with a Hamamatsu ORCAFlash-4.0LT (Hamamatsu, Naka-ku, Japan) CMOS camera mounted on an Olympus BX-60 fluorescence microscope (Tokyo, Japan) through a UPlanApo 100×/N.A. 1.35 oil Iris Ph3 objective. Images were acquired using the Micro Manager 1.4 plugin for ImageJ[16]. In all experiments, the cells were first photographed in phase contrast mode and then in fluorescence mode through filter cube EN-GFP (green, ex470/40, dic495LP, em525/50). Fluorescence backgrounds were subtracted using the modal values from the fluorescence images. Quantifications of cellular fluorescence were done using the ObjectJ plug-in of ImageJ[17].

1.11 Checkerboard synergy assay

As described by Hsieh *et al.*,[6] two-fold dilution series of cationic peptides were made in polypropylene microtiter plates (Ratiolab, L6018123, Dreieich, Germany) in 50 µL volume of MOPS medium/2% DMSO. Primary clinical isolates of *Escherichia coli* 87 were provided by Dr. Karin van Dijk, Amsterdam UMC. In a similar manner, two-fold dilution series of 17fα were made in polystyrene microtiter plates (Costar, REF3779, Corning NY, United States) in 70 µL volume of MOPS medium. Then, 50 µL of the 17fα series was transferred to the L8S1 containing polypropylene plate, resulting in a checkerboard titration of a L8S1 on the vertical axis and an 17fα on the horizontal axis. Mid-log phase cultures were diluted to OD_{600} 0.1 and 10 µL culture was added to each well of the microtiter plate, resulting in a total volume of 110 µL and an *OD*600 of ~0.01 (~3 × 108 CFU/mL), and the OD_{600} was measured every 15 min for 12 h using the microplate reader. First, the MIC values of the compounds alone were determined as described previously. Similarly, the MIC values of the compound combination were determined (the fractional inhibitory concentrations (*FIC*))[6]. With these *MIC* values and *FIC* values, the fractional inhibitory concentration index (FIC_{index}) was calculated as follows:

$$FIC_{index} = \frac{FIC_{antibiotic}}{MIC_{antibiotic}} + \frac{FIC_{peptide}}{MIC_{peptide}} \qquad (1)$$

$FIC_{\text{index}} \leq 0.5$ was considered synergistic, whereas $FIC_{\text{index}} \geq 2$ was considered antagonistic. An FIC_{index} between 0.5 and 1 was considered to be an additive effect.

1.12 Plasmid construction

All DNA manipulations were carried out according to standard procedures. In short, PCR amplification of the mScarlet gene and the kanamycin resistance gene were performed using High Fidelity DNA polymerase and plasmid DNA as template. Primers were flanked with Xho and BamHI (mScarlet sequence) and NheI and EarI (Kanamycin resistance sequence) restriction sites (see below). The resulting PCR fragments were cloned into the pRSF1010 plasmid, creating pRSF1010_kan-mScarlet. The plasmid was transformed to *E. coli* DH5α during the cloning process, after which it was isolated using the GeneJet Plasmid Miniprep Kit (Thermo Scientific, K0503, Waltham MA, United States). Next, isolated plasmid was first transformed to *E. coli* Nissle and isolated again to allow for further transformation to clinical isolate *E. coli* 87. Finally, the plasmid was transformed to *E. coli* 87, resulting in fluorescent mScarlet expressing *E. coli* 87.

**Primer ***	**Sequence (5’ > 3’) ****
mScarlet fw	gcatctcgagGATCTAAGCTGTTGTGACCGCTTGCT
mScarlet rv	GCTTTGTTAGCAGCCGGATCCTACTTGTACA
Kanamycin resistance fw	gGCTAGCgttgtgtctcaaaatctctgatgttacattgca
Kanamycin resistance rv	gcAGCGGAAGAGCccaattctgattagaaaaactcatcgagca

* fw: forward, rv: reverse

** restriction sites used for cloning are underlined

1.13 Microinjection of zebrafish larvae with *E.coli* 87 expressing mScarlet

Transparent Casper zebrafish (*Danio rerio*) larvae were infected and treated according to protocols described by Van der Sar *et al.* and Bernard *et al.*.[7,8] In brief, zebrafish larvae collected from a laboratory-breeding colony kept at 24 °C and a 12:12 h dark/light regime. Larvae were selected based on morphology according to hours post fertilization (hpf) and kept at 28 °C. All protocols followed the international guidelines on the protection of animals used for scientific purposes specified by the EU Directive 2010/63/EU, which allows zebrafish larvae to be used up to the moment of free-living (5–7 dpf). Larvae were dechorionated at 28 hpf and anaesthetized in 0.2% tricaine methanesulfonate (MS222, Fluka A-5040). Larvae were individually infected with 1 nL, containing approximately 150-200 CFU (1.5-2.0 CFU/mL), of mScarlet expressing clinical isolate *E. coli* 87 via microinjection of the caudal vein as described previously.[9] One hour post infection, larvae were treated with L8S1, 17fα or a combination of the two via another microinjection of 1 nL in the caudal vein. To correct dilution of the treatment upon injection in the vein (1 nL in 250 nL zebrafish larvae volume), treatments were diluted in 0.1% phenol red (Sigma-Aldrich, p-0290) in phosphate buffered saline (PBS) to 250x the concentration as tested *in vitro*. Zebrafish larvae were kept at 30 °C throughout the experiment and survival of the larvae, based on heartbeat, was determined every 24 h for 2 d. Moreover, 18 h post infection larvae were anaesthetized using MS222 and bacterial load was determined using an Olympus IX83 fluorescence microscope (4x objective magnification, Hamamatsu ORCA-Flash 4.0 camera) at specific wavelength for detection of red fluorescence (excitation/emission, 580/610 nm). Obtained images were analyzed using CellProfiler 3.19 (Broad Institute, Cambridge, MA, USA) with a custom-made automated analysis pipeline to count and quantify pixel intensity within the larvae. Integrated red fluorescence intensity per larvae was used as a quantification of bacterial burden.

2. Supporting tables

Table S1. Sequence and analytics of noncovalent inhibitors. Purity and *m*/*z*-values were determined via analytical RP-HPLC-MS with absorption at 210 nM. PG sequence element is a Fmoc-L-Pro-Gly-OH dipeptide (N-alpha-(9-Fluorenylmethyloxycarbonyl)-L-prolinyl-glycin, Iris-Biotech GmbH). LS sequence element is a Fmoc-L-Leu-L-Ser[PSI(Me,Me)Pro]-OH pseudo-proline ((S)-3-(N-(9-Fluorenylmethyloxycarbonyl)-L-leucinyl)-2,2-dimethyloxazolidine-4-carboxylic acid, Iris Biotech GmbH). B is Fmoc-B-OH (Norleucine: (S)-2-((((9H-fluoren-9-yl)methoxy)carbonyl)amino)hexanoic acid). Bold numbers represent non-natural amino acid building blocks with number equivalent amount of carbon atoms contributing to the final crosslink.

Peptide	**N-Term.**	**Sequence**	**Exact mass**	**$[M+2H]^{2+}$ Calc.**	***m/z* found**
24	FITC-PEG$_2$	QEALEERARNELSBTRPGETFYRL	3410.6	1706.3	1707.2
	AcN		2918.5	1460.3	1460.9
24a	FITC-PEG$_2$	QEALEERA**3**NELSBTRPG**3**TFYRL	3319.6	1660.8	1661.5
24b	FITC-PEG$_2$	QEALEERA**4**NELSBTRPG**3**TFYRL	3333.6	1667.8	1668.9
24c	FITC-PEG$_2$	QEALEERA**4**NELSBTRPG**5**TFYRL	3361.6	1681.8	1682.4
24d	FITC-PEG$_2$	QEALEERA**4**NELSBTRPG**6**TFYRL	3375.6	1688.8	1690.3
24e	FITC-PEG$_2$	QEALEERA**4**NELSBTRPG**7**TFYRL	3389.6	1695.8	1697.0
24f	FITC-PEG$_2$	QEALEERA**6**NELSBTRPG**6**TFYRL	3403.7	1702.9	1703.5
	AcN		2918.5	1456.8	1457.0
24g	FITC-PEG$_2$	QEALEERA**6**NELSBTRPG**7**TFYRL	3419.7	1709.8	1710.5

Table S2. Average distances ($\bar{d}$) in Ångström (Å) over 400 ns MD simulation between the Nε of three FtsQ binding site lysines and Cβ of selected 24f residues.

		Nε		
Cβ		**K208**	**K218**	**K239**
64	Q	18.4	25.4	28.0
65	E	13.9	21.4	25.2
66	A	19.1	20.5	29.4
67	L	20.7	22.8	27.9
68	E	16.3	20.6	22.8
69	E	17.5	16.0	25.1
70	R	22.4	18.2	28.1
71	A	21.3	20.2	24.0
72	**R**			
73	N	23.2	13.8	26.1
74	E	26.0	18.1	26.6
75	L	24.5	20.2	22.8
76	S	24.1	19.3	19.2
77	B	21.8	22.0	18.7
78	T	19.7	23.2	14.4
79	R	16.8	25.3	15.2
80	P			
81	G			
82	**E**			
83	T	18.3	24.3	7.5
84	F	19.9	20.2	12.9
85	Y	20.1	18.9	11.8
86	R	24.2	16.5	16.9
87	L	24.1	14.0	18.6

Table S3. Sequence and analytics of covalent inhibitors. Purity and mass-to-charge ratio was determined via analytical RP-HPLC-MS with absorption at 210 nm. PG sequence element is a Fmoc-L-Pro-Gly-OH dipeptide (N-alpha-(9-Fluorenylmethyloxycarbonyl)-L-prolinyl-glycin, Iris-Biotech GmbH). LS sequence element is a Fmoc-L-Leu-L-Ser[PSI(Me,Me)Pro]-OH pseudo-proline ((S)-3-(N-(9-Fluorenylmethyloxycarbonyl)-L-leucinyl)-2,2-dimethyloxazolidine-4-carboxylic acid, Iris Biotech GmbH). B is Fmoc-B-OH (norleucine: (S)-2-((((9H-fluoren-9-yl)methoxy)carbonyl)amino)hexanoic acid) and m represents the site of modifier incorporation via Fmoc-Lysine(MMT)-OH that is used for the implementation of modifier (Figure S3). Bold numbers represent non-natural amino acid building blocks with number equivalent amount of carbon atoms contributing to the final crosslink (Supporting Figure S1).

Peptide	**Mod.**	**N-Term.**	**Sequence**	**Exact mass**	**$[M+2H]^{2+}$ Calc.**	***m/z* found**
24	AcN	FITC-PEG$_2$	QEALEERARNELSBTRPGEmFYRL	3479.7	1740.8	1741.8
	α	AcN		3065.5	1533.7	1535.2
	κ	AcN		3021.5	1511.8	1512
	χ	AcN		2999.6	1500.8	1001.1
	β	AcN		3077.5	1539.7	1540.4
	ε	AcN		3033.5	1517.8	1518.2
	ϕ	AcN		3071.6	1537.7	1537.5
	ι	AcN		3056.6	1529.3	1529.9
	η	AcN		3131.5	1566.8	1567.2
	γ	AcN		3139.6	1570.8	1571.0
	φ	AcN		3106.5	1554.3	1554.5
	δ	AcN		3092.5	1547.2	1547.9
24f	α	AcN	QEALEERA**6**NELSBTRPG**6**mFYRL	3058.5	1530.3	1531.3
	β	AcN		3070.5	1536.3	1537.4
	γ	AcN		3132.6	1567.3	1568.3
	δ	AcN		3088.4	1543.8	1541.0
20f	α	AcN	EERA**6**NELSBTRPG**6**mFYRL	2619.9	873.4	871.3
17f	α	AcN	A**6**NELSBTRPG**6**mFYRL	2203.12	1102.1	1103.7
	β	AcN		2215.12	1108.6	1109.6
	γ	AcN		2277.20	1139.6	1139.3
	δ	AcN		2230.13	1116.1	1116.7
15f	α	AcN	A**6**NELSBTRPG**6**mFY	1933.93	968.0	968.8

Table S4. Table of abundances in MS/MS experiment. Absolute abundance of FtsQ sequences in sample of unmodified and modified FtsQ as well as their ratio. Sequences with at least two counts were considered. Grey sequences were combined to one corresponding sequences (ID 3, 4, 5, 6, 9, 10 and 11). * represents an oxidized methionine.

		abundance			
ID	**Sequence Stretch**	**FtsQ(50-276)**	**+24fα**	**total ab.**	**ratio**
1	LVLTGER	36	42	78	1.17
2	HYTRNDDIR	5	8	13	1.60
3	QSILALGEPGTFMTQDVNIIQTQIEQR(*)	51	22	73	0.43
	QSILALGEPGTFMTQDVNIIQTQIEQR	19	-		
	QSILALGEPGTFMTQDVNIIQTQIEQR*	32	22		
4	K/QWPDELK/IHLVEYVPIAR	105	106	211	1.01
	KQWPDELK	19	25		
	QWPDELKIHLVEYVPIAR	7	3		
	IHLVEYVPIAR	79	78		
5	TSKQVLPMLYGPEGSANEVLQGYR(*)	10	12	22	1.20
	TSKQVLPMLYGPEGSANEVLQGYR	5	4		
	TSKQVLPMLYGPEGSANEVLQGYR*	5	8		
6	QVLPMLYGPEGSANEVLQGYR/EMGQMLAK(*)	200	200	400	1.00
	QVLPMLYGPEGSANEVLQGYR	47	52		
	QVLPMLYGPEGSANEVLQGYR*	43	52		
	QVLPMLYGPEGSANEVLQGYREMGQMLAK*	50	34		
	EMGQMLAK*	60	62		
7	DRFTLK	35	36	71	1.03
8	EAAMTAR*	13	8	21	0.62
9	R/SWQLTLNNDIK	127	144	271	1.13
	RSWQLTLNNDIK	66	82		
	SWQLTLNNDIK	61	62		
10	LNLGR/GDTMK/R	9	5	14	0.56
	LNLGRGDTMK	4	5		
	GDTMKR	5	-		
11	FVELYPVLQQQAQTDGK/R	128	25	153	0.20
	FVELYPVLQQQAQTDGK	41	5		
	FVELYPVLQQQAQTDGKR	87	20		
12	ISYVDLR	84	104	188	1.24
13	YDSGAAVGWAPLPPEESTQQQNQAQAEQQ	15	37	52	2.47

Table S5. Survival rates of zebrafish larvae depending on indicated treatment regime after 24 h. All measurements were performed in quadruplicate (*n* = 4 replicates, 15 zebrafish per condition, error = SD).

	**DMSO (50%)**	***E. coli* 87 (189 CFU)**	**+ L8S1 (3.125 µM)**	**+ 17fα (70 µM)**	**+ L8S1 + 17fα**
#1	100	13	20	0	33
#2	87	40	31	40	71
#3	100	20	27	20	47
#4	100	27	27	20	53
Mean	96.8	25.0	26.3	20.0	51.0
SD	6.5	11.5	4.6	16.3	15.7
Survival	96.8 ±6.5	25.0 ±11.5	26.3 ±4.6	20.0 ±16.3	51.0 ±15.7

Table S6. Toxicity test with 17fα, L8S1 and a combination of both. Surviving zebrafish larvae depending on indicated treatment regime over the course of two days. Measurements were performed once (n = 1 replicates, 15 zebrafish per condition).

	**Surviving zebrafish**	
	**Day 1**	**Day 2**
Untreated	15	15
DMSO (50%)	15	15
L8S1 (3.125 µM)	15	15
17fα (70 µM)	15	15
L8S1 + 17fα	15	15

3. Supporting figures

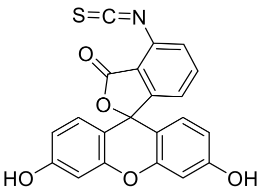

FITC
Fluorescein isothiocyanate

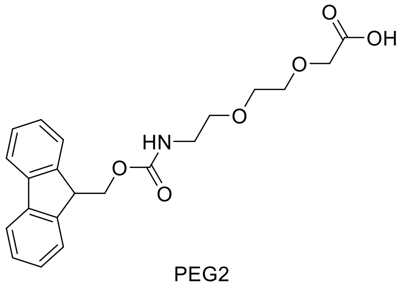

PEG2
1-(9*H*-fluoren-9-yl)-3-oxo-2,7,10-trioxa-4-azadodecan-12-oic acid

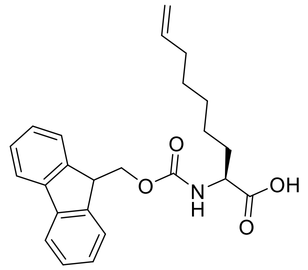

6
(*S*)-2-((((9*H*-fluoren-9-yl)methoxy)carbonyl)amino)-
non-8-enoic acid

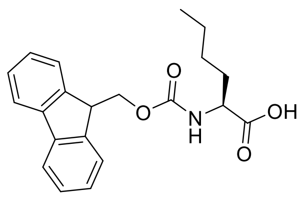

B
(*S*)-2-((((9*H*-fluoren-9-yl)methoxy)carbonyl)amino)-
hexanoic acid

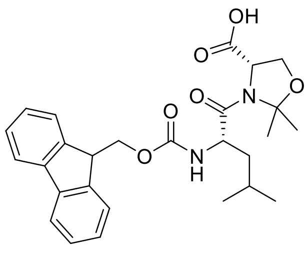

LS
Fmoc-L-Leu-L-Ser[PSI(Me,Me)Pro]-OH
(*S*)-3-((((9*H*-fluoren-9-yl)methoxy)carbonyl)-*L*-leucyl)-
2,2-dimethyloxazolidine-4-carboxylic acid

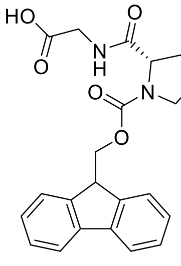

PG
Fmoc-L-Pro-Gly-OH
(((9*H*-fluoren-9-yl)methoxy)carbonyl)-
L-prolylglycine

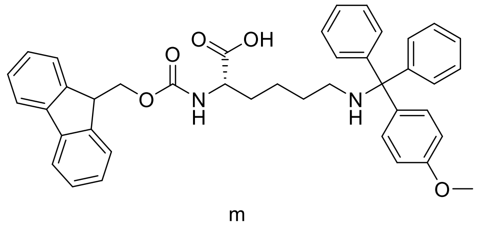

m
Fmoc-L-Lys(MMT)-OH
N-α-Fmoc-N-ε-4-methoxytrityl-L-lysine

Figure S1. Structure of FITC (fluorescein isothiocyanate), PEG_2 (1-(9H-fluoren-9-yl)-3-oxo-2,7,10-trioxa-4-azadodecan-12-oic acid), Fmoc-6-OH ((S)-2-((((9H-fluoren-9-yl)methoxy)carbonyl)amino)non-8-enoic acid), Fmoc-B-OH (norleucine: (S)-2-((((9H-fluoren-9-yl)methoxy)carbonyl)amino)hexanoic acid), Fmoc-L-Pro-Gly-OH (PG: N-alpha-(9-fluorenylmethyloxycarbonyl)-L-prolinyl-glycin), Fmoc-L-Leu-L-Ser[PSI(Me,Me)Pro]-OH (LS: (S)-3-(N-(9-fluorenylmethyloxycarbonyl)-L-leucinyl)-2,2-dimethyloxazolidine-4-carboxylic acid) and Fmoc-L-Lys(MMT)-OH (m, N-α-Fmoc-N-ε-4-methoxytrityl-L-lysine).

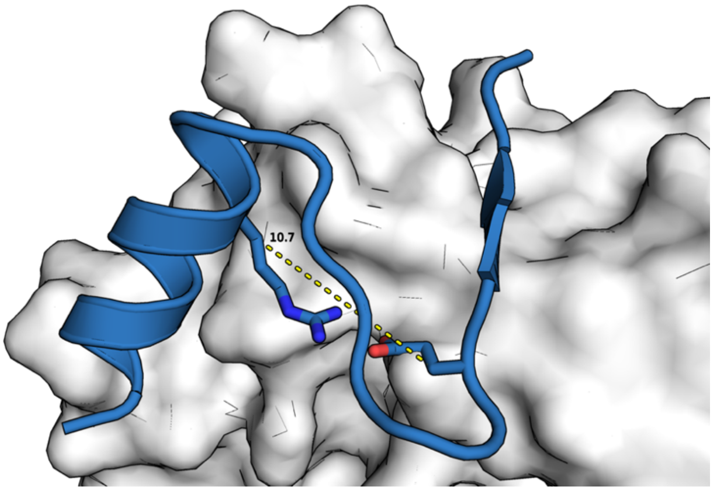

# C atoms	r (Å)
6	6.2
7	7.2
9	9.6
10	11.0
11	12.1
12	13.5
13	14.6

Figure S2. Left: Crystal structure (PDB: 6h9o) of FtsQ (grey) and 24 (blue) with distance in Å between Cβ of R72 and E82 in 24. Right: Distance between terminal carbon atoms of linear hydrocarbon chains with specified number of carbon atoms and a double bond in trans configuration at the central position. Reported distances (r) were obtained after energy minimization of these structures starting from their most extended conformation using the MOE2020.09 software[18] with the AMBER99 force field.[19]

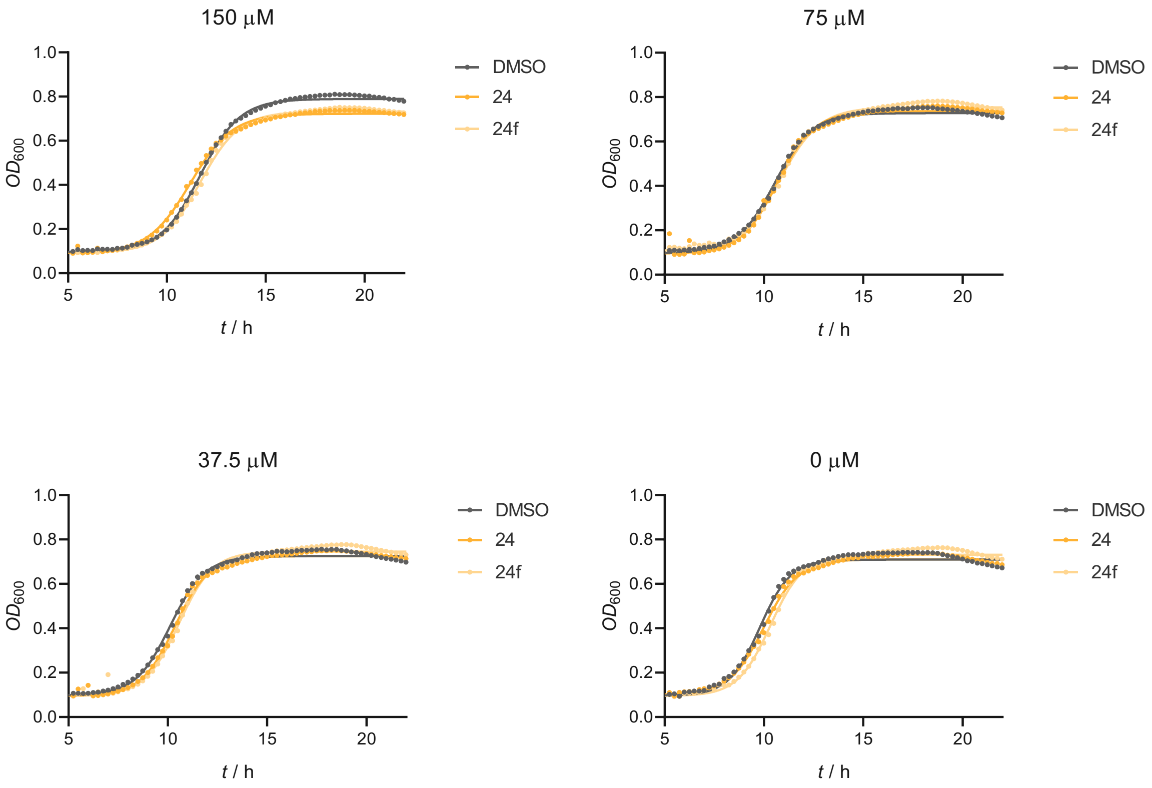

Figure S3. Growth assay results of 24 and stapled peptide 24f treated *E.coli lptD*4213 (imp) (*n* = 1 replicate). Corresponding peptide sequences are listed in Supporting Table S1.

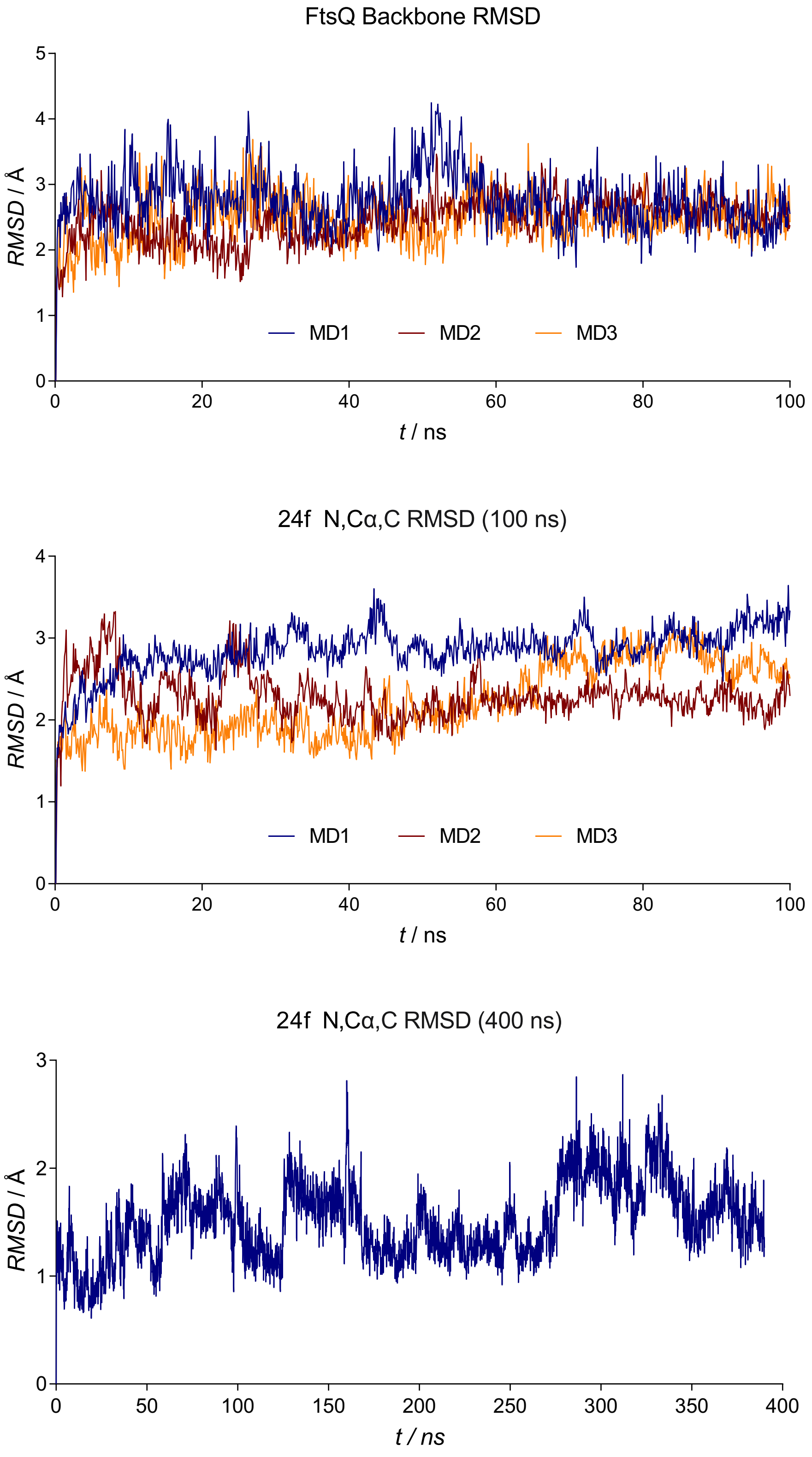

Figure S4. Top two: RMSD values of the FtsQ backbone and 24f Cα's obtained from three 100 ns MD simulations. Bottom: RMSD values of 24f Cα's obtained from a 400 ns MD simulations.

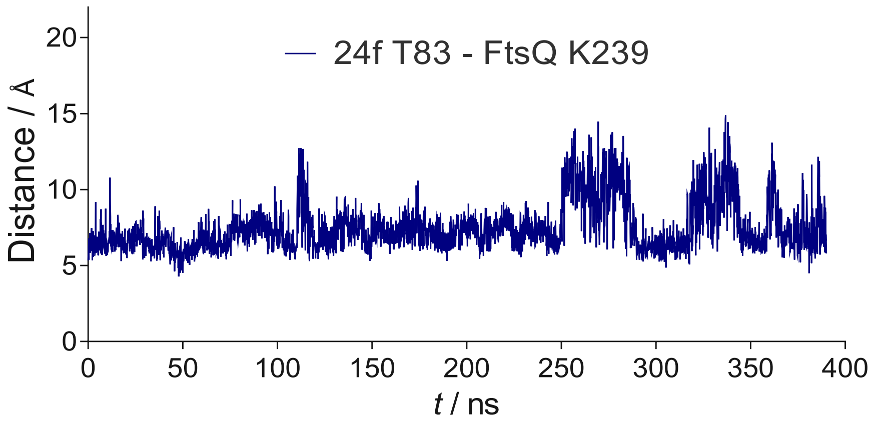

Figure S5. Distance of Nε of FtsQ lysines K239 to the Cβ of the corresponding T83 in 24f over a 400 ns MD simulation.

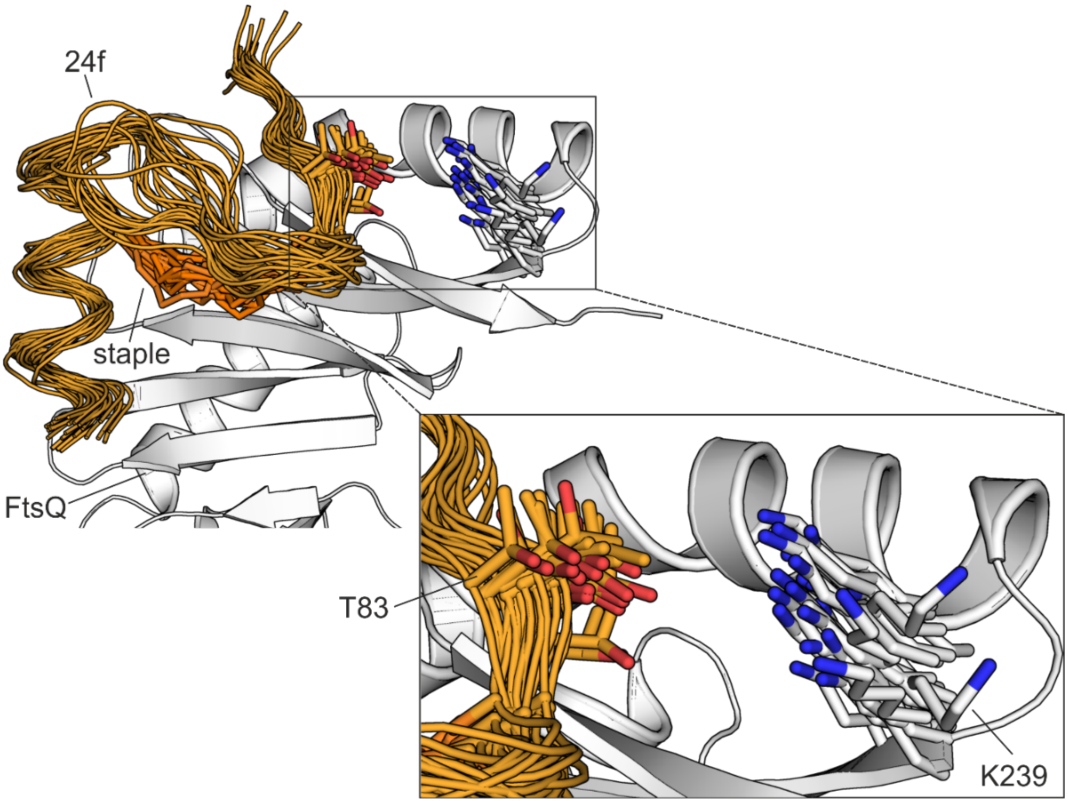

Figure S6. MD simulation snapshots (every 10 ns) over 100 ns time span in three separate MDs of 24f and FtsQ with focus on 24f T83 and FtsQ K239.

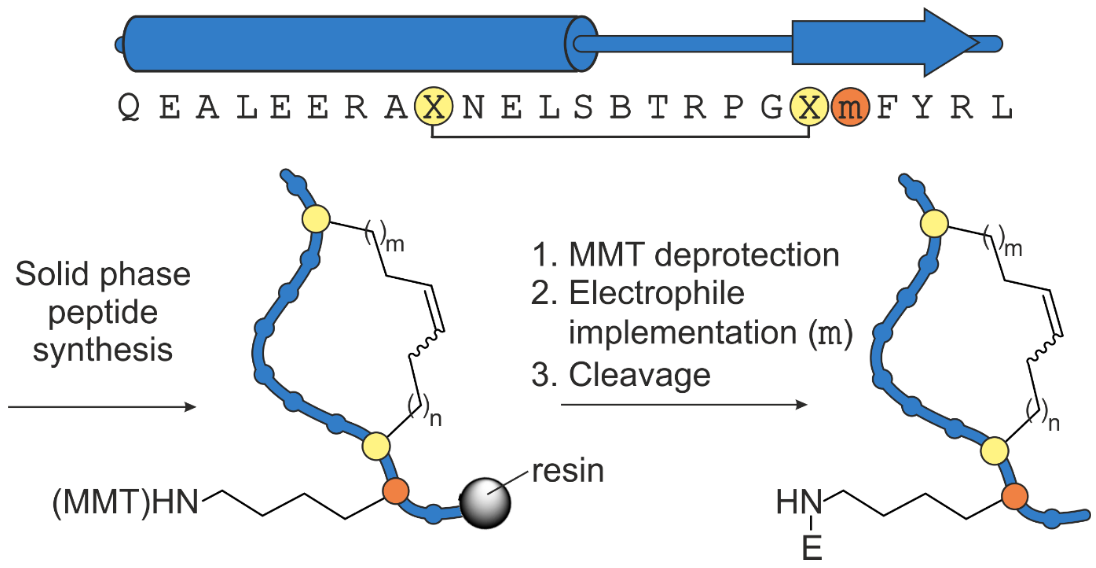

Figure S7. Synthesis scheme showing the introduction of modifiers via amid formation or nucleophilic aromatic substitution to obtain covalent inhibitors. For modifier implementation an Fmoc-protected lysine with a monomethoxytrityl (MMT) protected sidechain m (N-alpha-(9-Fluorenylmethyloxycarbonyl)-N-epsilon-4-methoxytrityl-L-lysine) (Supporting Figure S1) was incorporated during peptide synthesis. After RCM and N-terminal modification the MMT protected Lysine sidechain was selectively deprotected (1% TFA and 2% TIPS in DCM). Modifiers bearing a carboxylic acid handle (4 eq.) were implemented using COMU/Oxyma (4eq.) and DIPEA (4eq.) in DMF. Modifier δ (2,4,6-trichloro-1,3,5-triazine) (4 eq.) was coupled using DIPEA (8eq.) in THF.

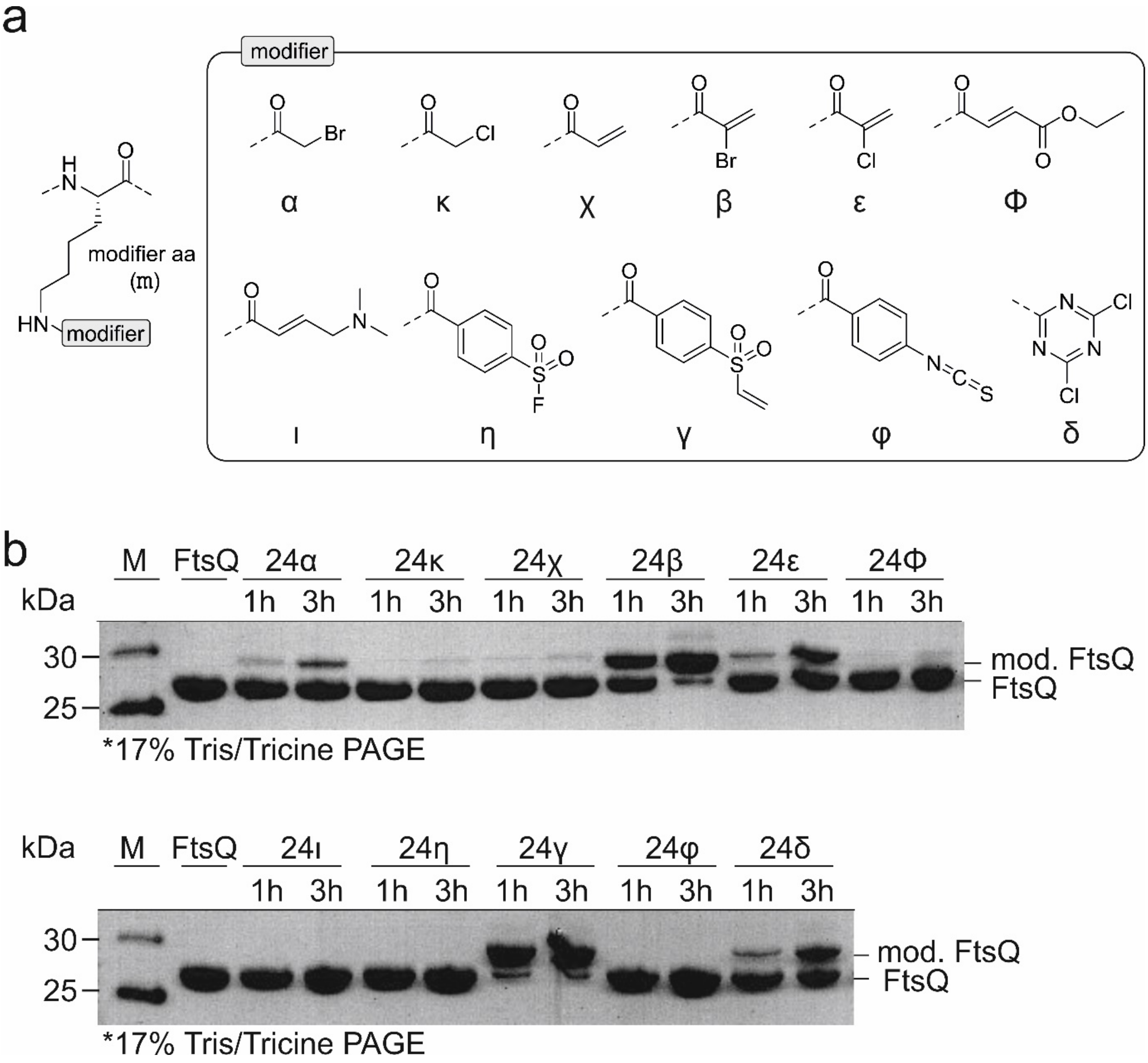

Figure S8. (**a**) Set of modifiers tested in Protein Modification Assay using peptide 24 as scaffold. Reaction details are described in section 1.1.3. α: 2-bromoacetic acid,[20] κ: 2-chloroacetic acid,[20,21] χ: acrylic acid,[20–24] β: 2-bromoacrylic acid, ε: 2-chloroacrylic acid, φ: (*E*)-4-ethoxy-4-oxobut-2-enoic acid,[25] ι: (*E*)-4-(dimethylamino)but-2-enoic acid,[20–24] η: 4-(fluorosulfonyl)benzoic acid,[24,26–29] γ: 4-(vinylsulfonyl)benzoic acid,[22,23,30] φ: 4-isothiocyanatobenzoic acid,[31] δ: 2,4,6-trichloro-1,3,5-triazine,[23,25,29,31] (**b**) Protein Modification Assay with 24-based electrophilic peptides. FtsQ(50-276) (c = 50 µM) was incubated with electrophilic peptide (c = 125 µM) in 20 mM NH_4HCO_3 buffer (pH 7.6, 150 mM NaCl) at 37 °C for the given time period (1h or 3h).

a

```
FtsQ(50-276)

GHHHHHHEDA QRLPLSKLVL TGERHYTRND DIRQSILALG EPGTFMTQDV
NIIQTQIEQR LPWIKQVSVR KQWPDELKIH LVEYVPIARW NDQHMVDAEG
NTFSVPPERT SKQVLPMLYG PEGSANEVLQ GYREMGQMLA KDRFTLKEAA
MTARRSWQLT LNNDIKLNLG RGDTMKRLAR FVELYPVLQQ QAQTDGKRIS
YVDLRYDSGA AVGWAPLPPE ESTQQQNQAQ AEQQ
```

b

FtsQ(50-276)	calc. MW	26939.4
	found	26943.0

Signal	$[M+xH]^{x+}$	m/z calc.	m/z found
1	38	709.9	710.1
2	37	729.1	729.5
3	36	749.3	749.6
4	35	770.7	770.4
5	34	793.3	793.1
6	33	817.3	817.5
7	32	842.9	842.5
8	31	870.0	869.8
9	30	899.0	899.1
10	29	929.9	930.1
11	28	963.1	962.8
12	27	998.8	997.5
13	26	1037.1	1037.3
14	25	1078.6	1078.3
15	24	1123.5	1123.4
16	23	1172.3	1172.2
17	22	1225.5	1225.5

FtsQ(50-276)-17fα	calc. MW	29061.6
	found	29066.8

Signal	$[M+xH]^{x+}$	m/z calc.	m/z found
1	40	727.5	728.4
2	39	746.2	746.3
3	38	765.8	765.5
4	37	786.4	786.9
5	36	808.3	808.7
6	35	831.3	831.5
7	34	855.8	855.8
8	33	881.7	881.3
9	32	909.2	909.1
10	31	938.5	938.8
11	30	969.7	970
12	29	1003.1	1003
13	28	1038.9	1039.1
14	27	1077.4	1077.5
15	26	1118.8	1118.7
16	25	1163.5	1163.8
17	24	1211.9	1212.8
18	23	1264.5	1265.2

c

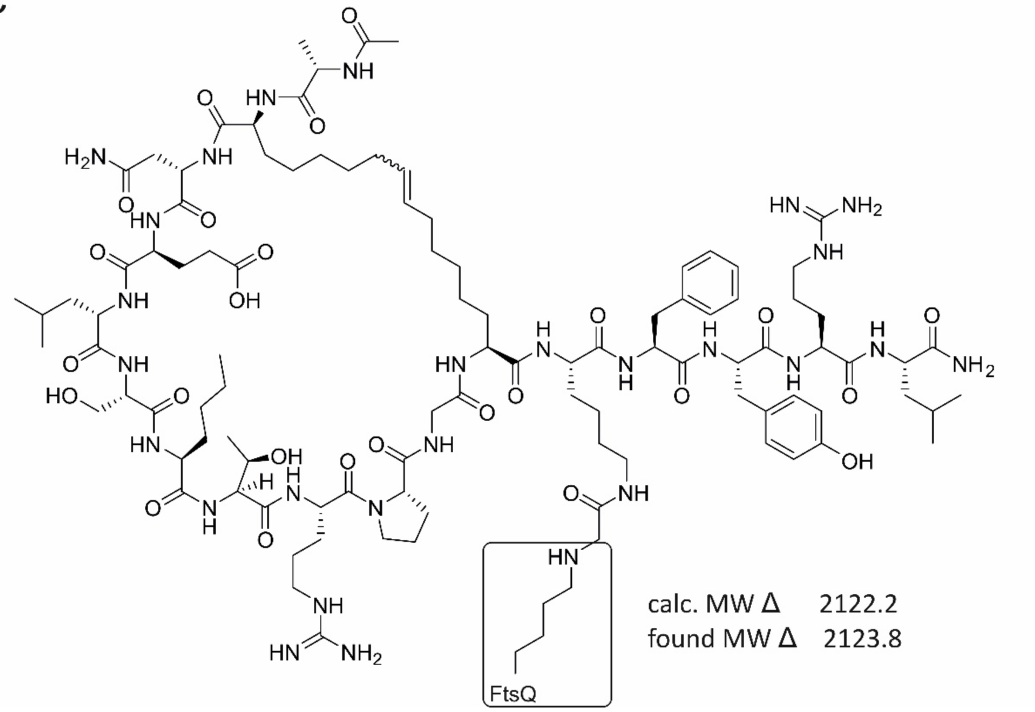

Figure S9. HPLC/MS analysis of FtsQ(50-276) incubated for 3 h at 37°C in binding buffer (20 mM NH_4HCO_3 buffer, pH 7.6, 150 mM NaCl) with corresponding ESI-MS assignment including calculated and found *m*/*z*-values.

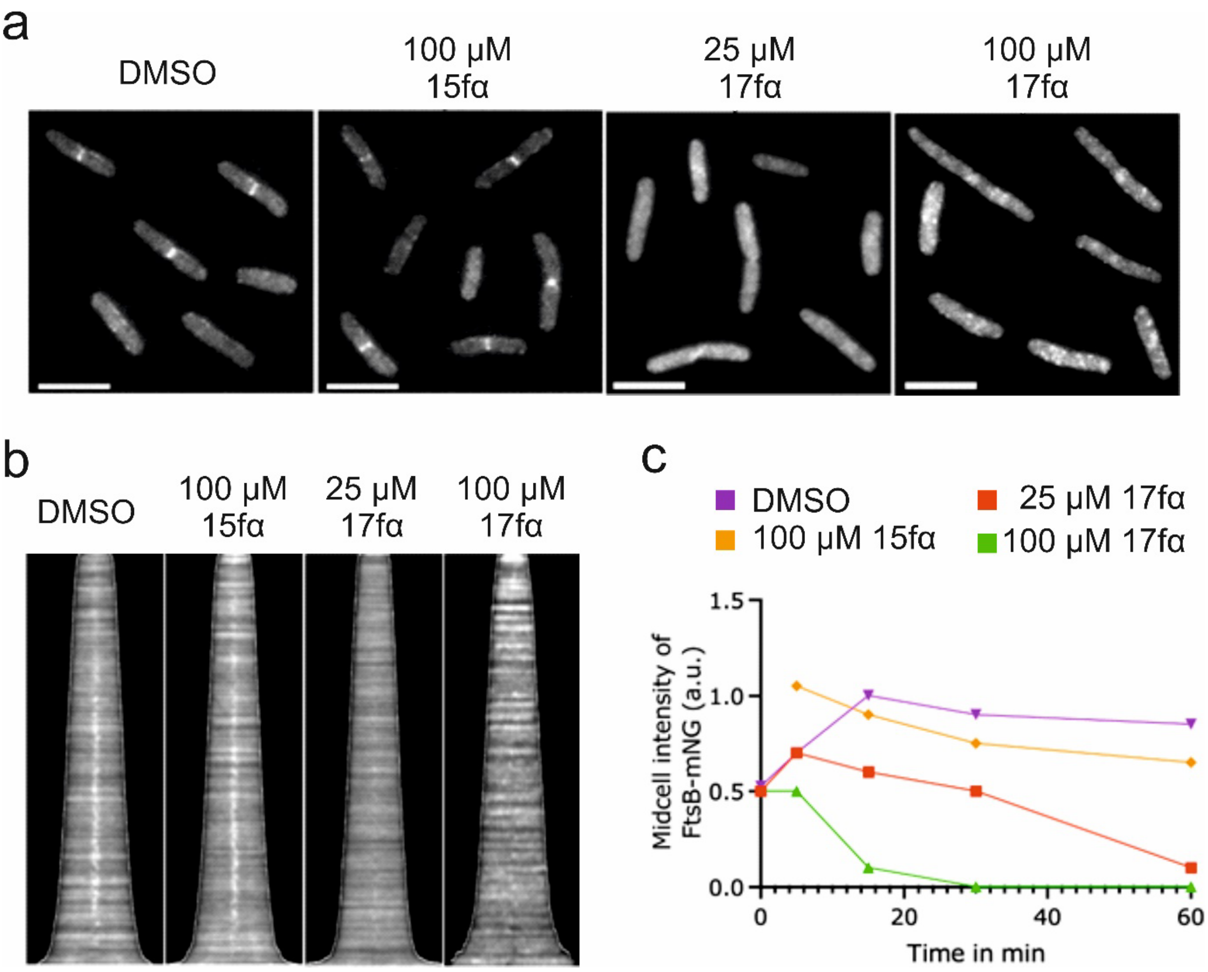

Figure S10. FtsB mimicking peptide inhibits mid cell localization of FtsB-mNG. (**a**) Representative cells of the indicated samples after 60 min of incubation (scale bar: 5 µm). (**b**) Demograph of the localization of FtsB-mNG in cells sorted according to their cell length after 60 min of incubation with the indicated compounds. This was performed using the ObjectJ plug-in of ImageJ.[17] The tool measures the Fluorescence intensity along the longitudinal axis of individual cells relative to its cell length and then integrates across all recorded measurements. (**c**) The amount of FtsB-mNG present at midcell in comparison to the amount in the cylindrical part of the cells as function of incubation time while growing in MOPS rich medium at 37 °C. *LptD*4213 FtsB-mNG in the presence of 0.33% DMSO (Orange), of 100 µM negative control peptide 15fα (purple), of 25 µM inhibitory peptide 17fα (red) or of 100 µM inhibitory peptide 17fα (green).

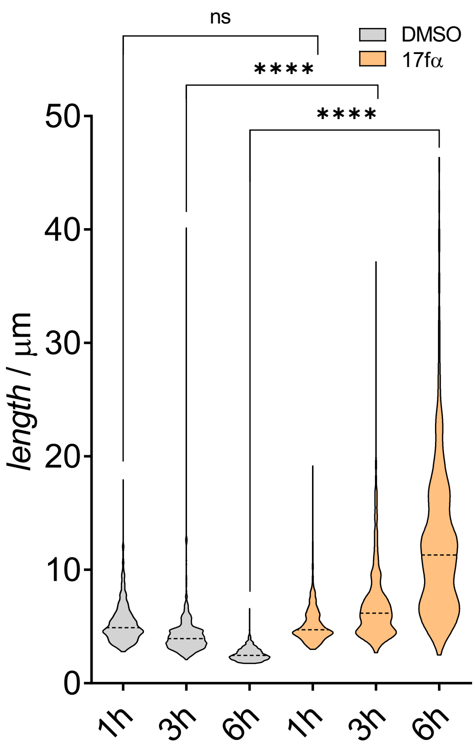

Figure S11. Violin plot of cell length distribution of *E.coli LptD*4213 treated with either DMSO or 100 µM 17fα after 1h, 3h and 6h. Significance was determined by Kruskal-Wallis test and Dunn's multiple comparisons test.

a

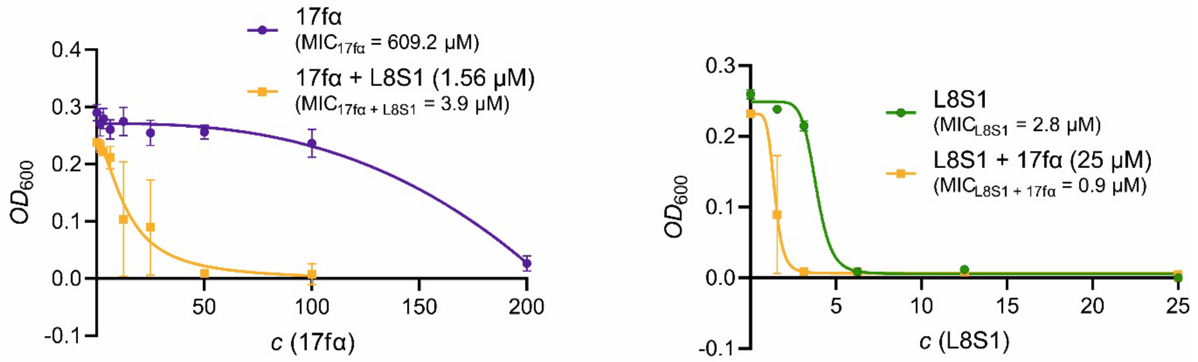

b

	MIC value	*FIC* value
17fα	609.2	
17fα + L8S1 (1.56 µM)	3.9	0.01
L8S1	2.8	
L8S1 + 17fα (µM)	0.9	0.032

c

$$FIC_{\text{index}} = \frac{FIC_{17f\alpha}}{MIC_{17f\alpha}} + \frac{FIC_{L8S1}}{MIC_{L8S1}} = 0.33$$

Figure S12. *In vitro* synergy assay results of stapled covalent inhibitor 17fα and potentiator L8S1 treated *E.coli* 87. (**a**) OD_{600} of samples after 12 h (Checkerboard synergy assay: $n = 3$ replicates, error = SEM). (**b**) *MIC* value was determined as the concentration at which 90% of bacterial growth was inhibited as determined by nonlinear regression analysis (L8S1: $n = 3$ replicates, 17fα: $n = 5$ replicates). (**c**) The *FIC* was determined from the averages of the *MICs*.

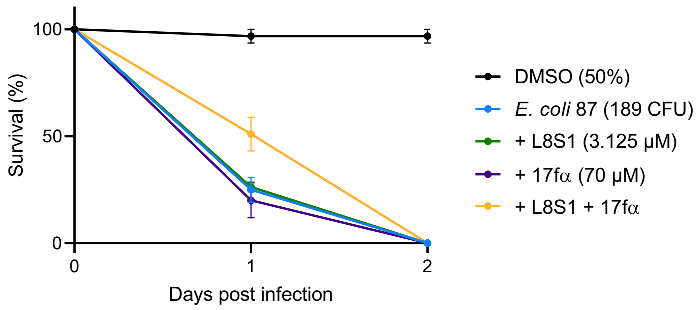

Figure S13. Survival rates of zebrafish larvae depending on indicated treatment regime over the course of two days. All measurements were performed in quadruplicate (n = 4 replicates, 15 zebrafish per condition, error bar = SEM).

Replicates 1-4:

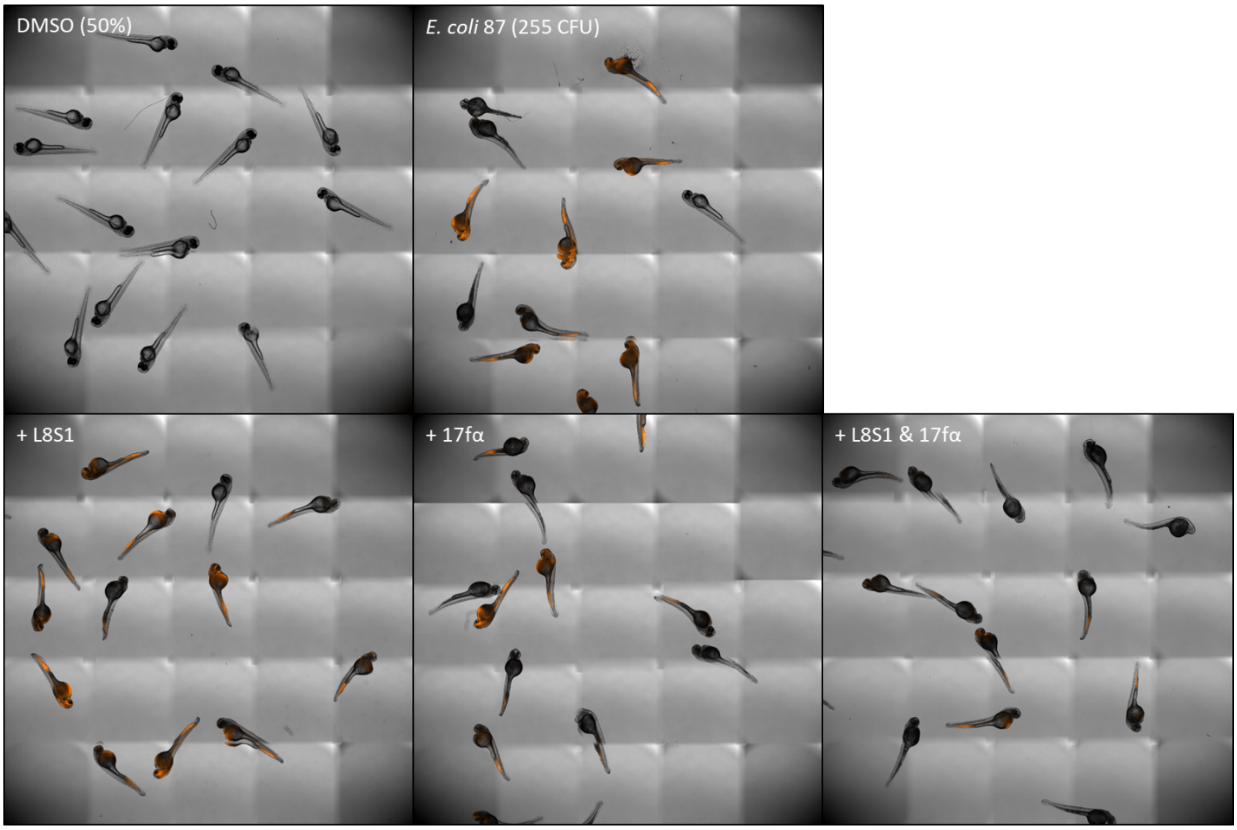

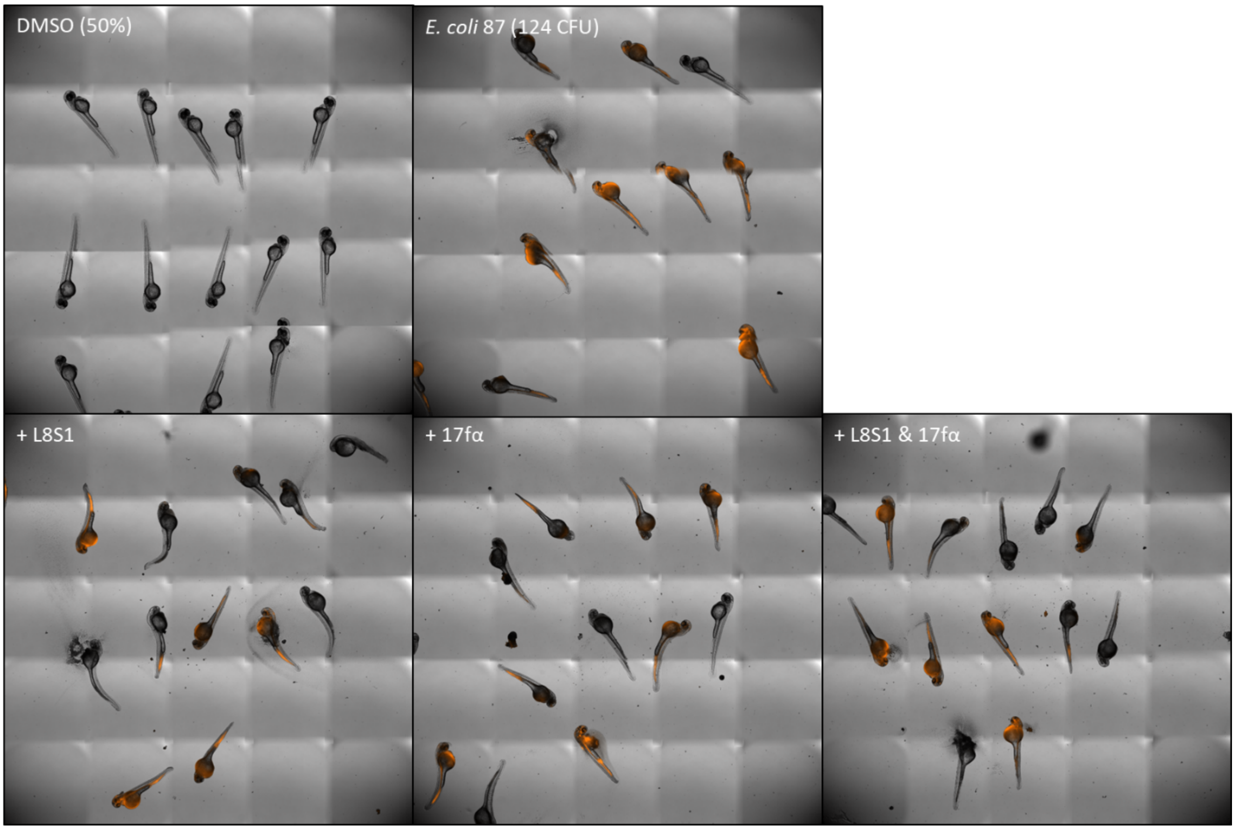

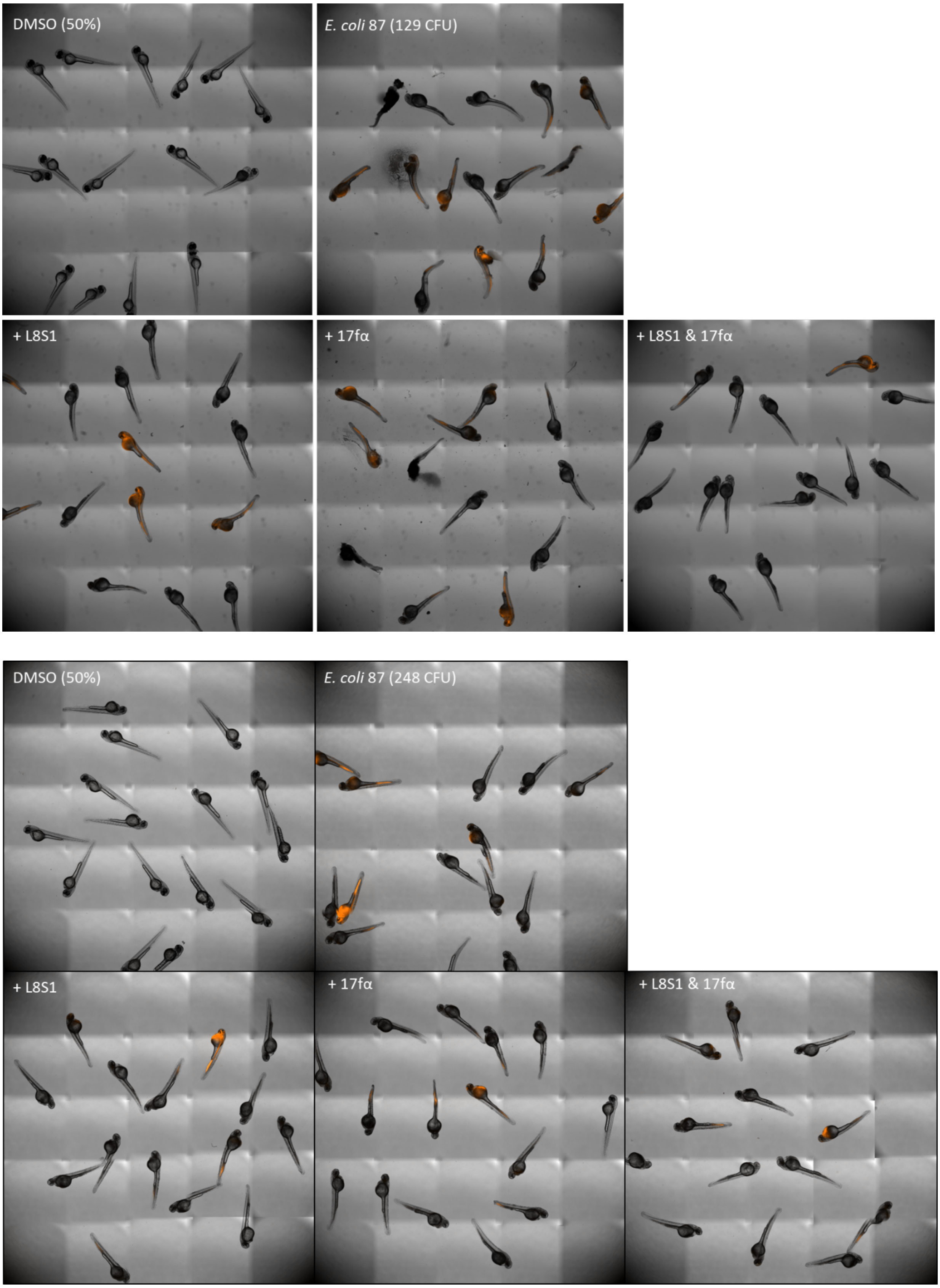

Figure S14. Replicates of Zebrafish Infection assay: Pictures of Zebrafish larvae infected with *E.coli* 87 (+ DMSO control) encoding fluorescent mScarlet treated with indicated compounds.

4. Peptide structures and analytics

FITC-PEG$_2$-24

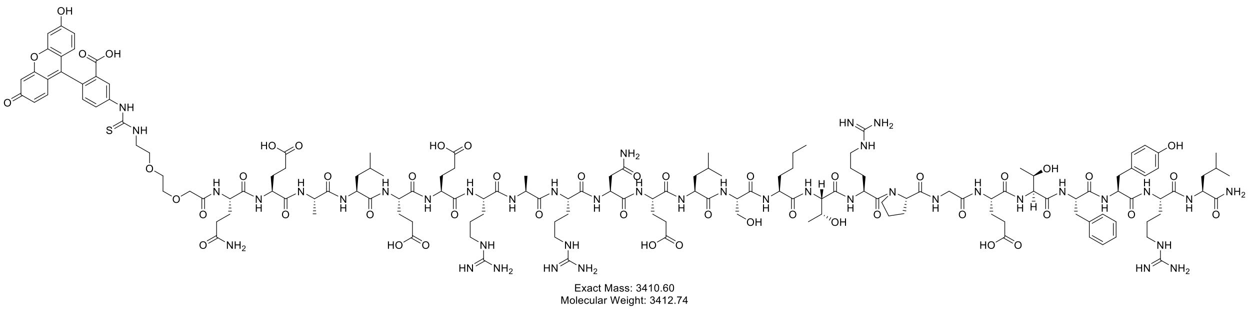

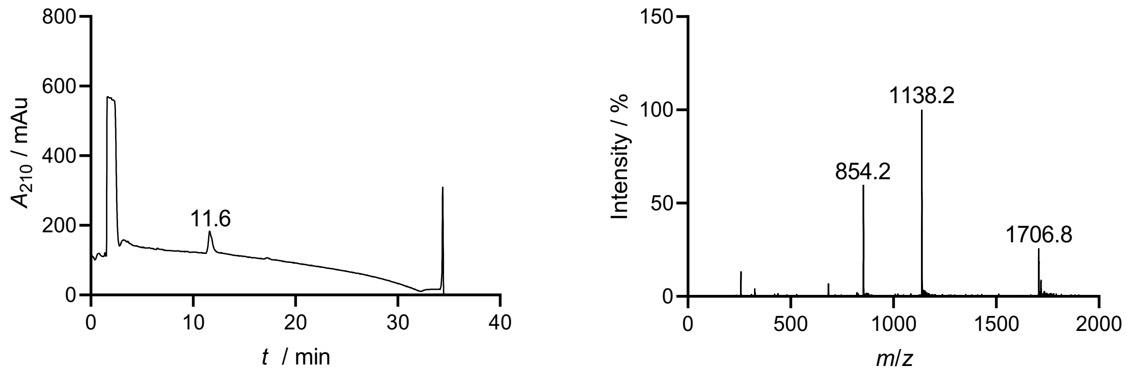

Left: HPLC chromatogram at λ = 210 nm, peak retention time: 11.6 min, gradient: 5–95 % ACN in 30 min; Right: MS spectrum, *MW*: 3412.74 g·mol^{-1}, calc. *m*/*z*: 1706.3 / 1138.9 / 853.7.

24

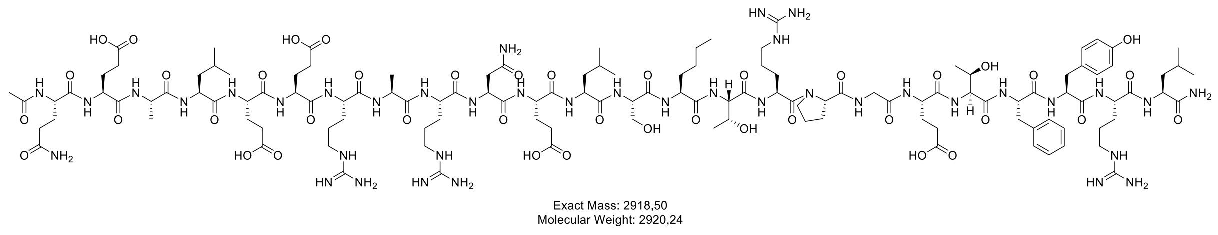

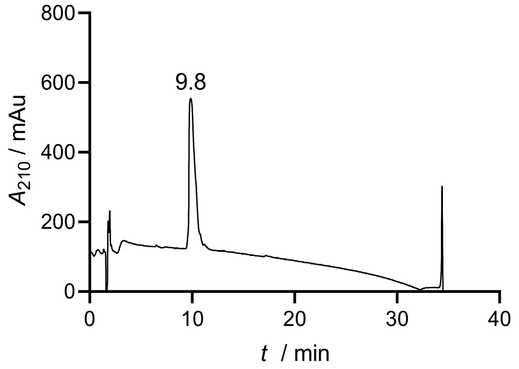

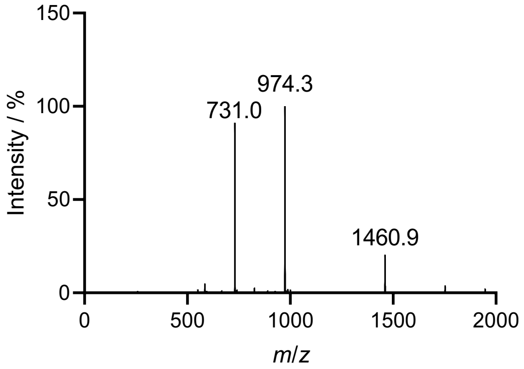

Left: HPLC chromatogram at λ = 210 nm, peak retention time: 9.8 min, gradient: 5–95 % ACN in 30 min; Right: MS spectrum, *MW*: 2920.24 g·mol^{-1}, calc. *m*/*z*: 1460.3 / 973.8 / 730.6.

FITC-PEG$_2$-24f

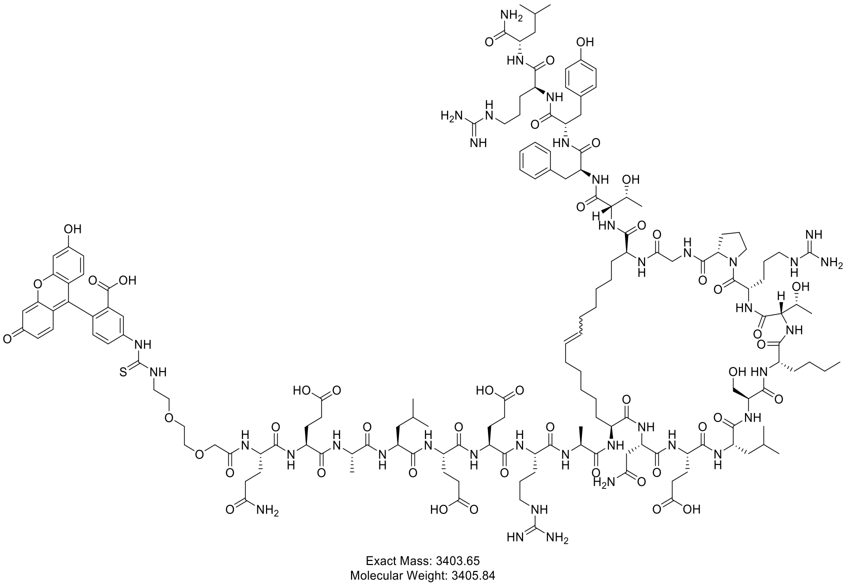

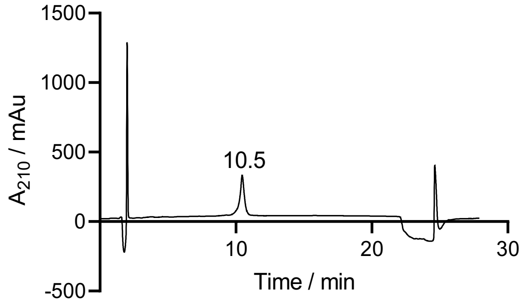

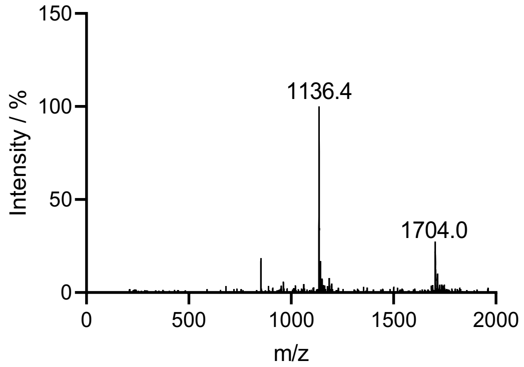

Left: HPLC chromatogram at $\lambda = 210$ nm, peak retention time: 10.5 min, gradient: 30–60 % ACN in 20 min; Right: MS spectrum, *MW*: 3405.84 g·mol^{-1}, calc. *m*/*z*: 1702.9 / 1135.6.

24f

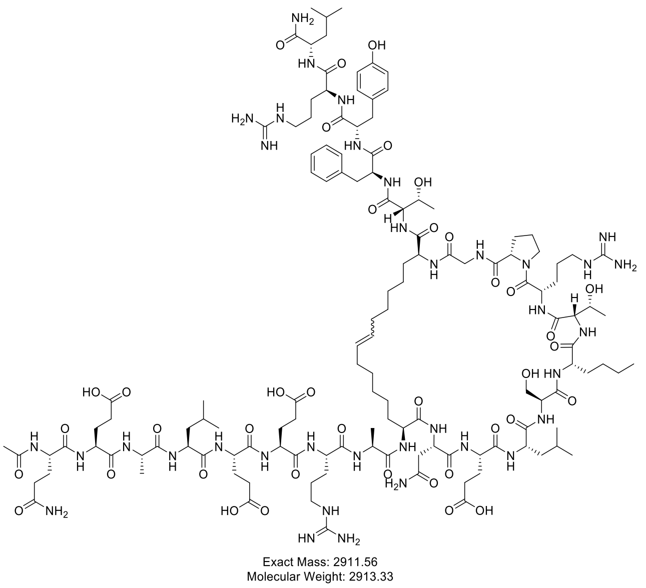

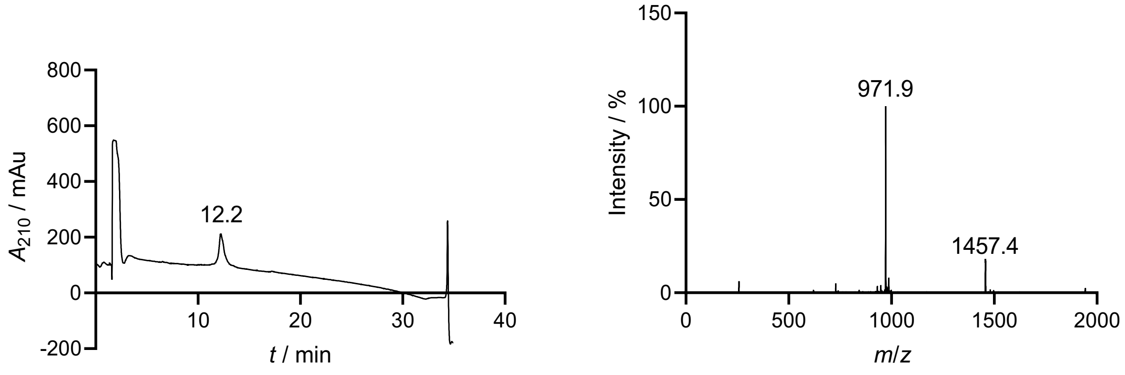

Left: HPLC chromatogram at λ = 210 nm, peak retention time: 12.2 min, gradient: 5–95 % ACN in 30 min; Right: MS spectrum, *MW*: 2913.33 g·mol^{-1}, calc. *m*/*z*: 1456.8 / 971.5.

24α

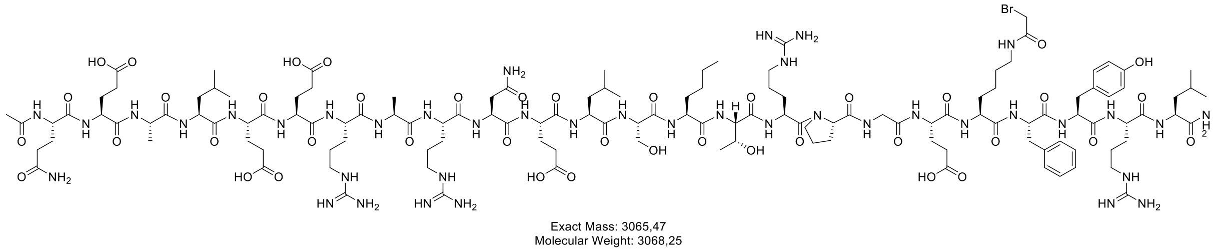

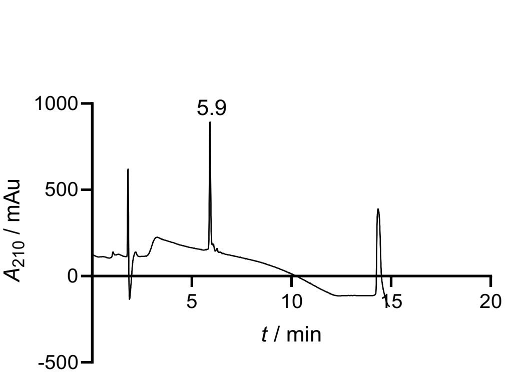

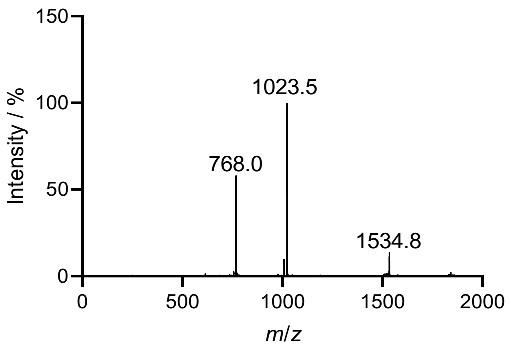

Left: HPLC chromatogram at λ = 210 nm, peak retention time: 5.9 min, gradient: 5–95 % ACN in 10 min; Right: MS spectrum, *MW*: 3068.25 g·mol^{-1}, calc. *m*/*z*: 1533.74 / 1022.82.

24β

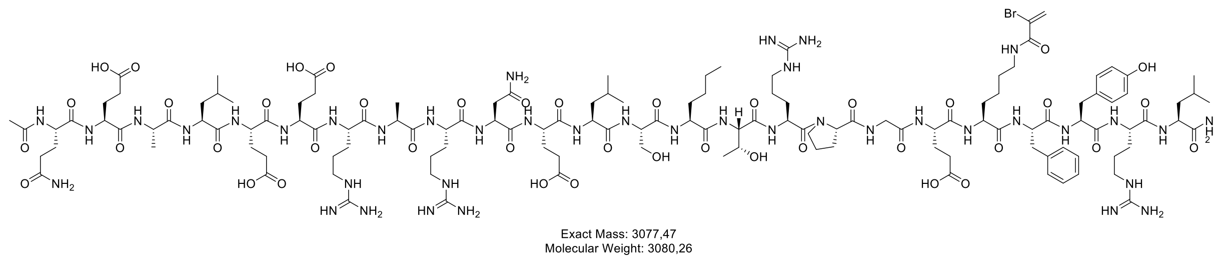

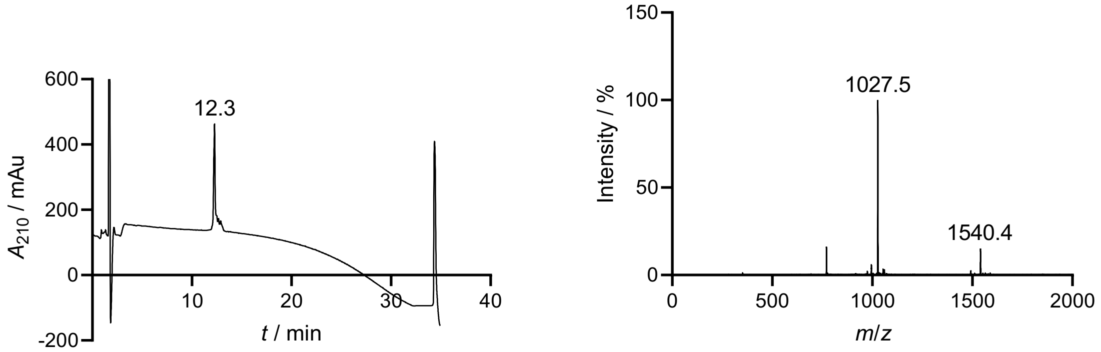

Left: HPLC chromatogram at $\lambda = 210$ nm, peak retention time: 12.3 min, gradient: 5–95 % ACN in 30 min; Right: MS spectrum, *MW*: 3080.26 g·mol^{-1}, calc. *m*/*z*: 1539.74 / 1026.82.

24ε

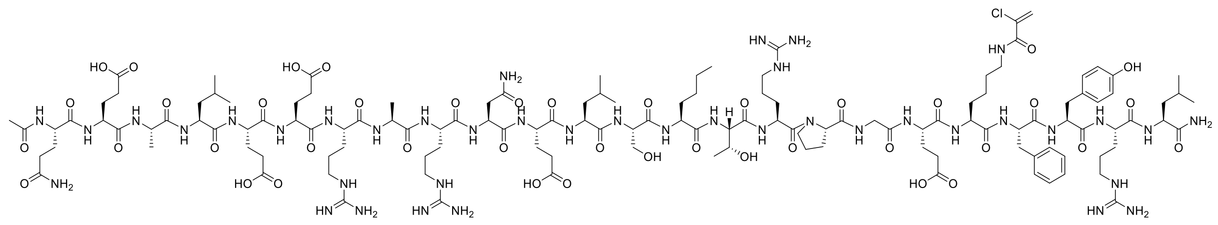

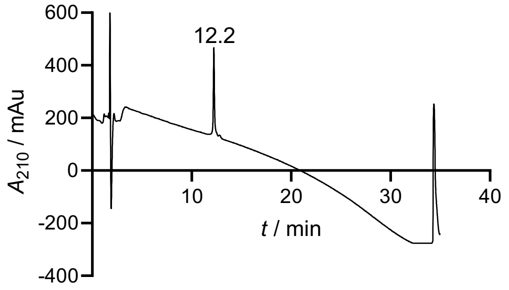

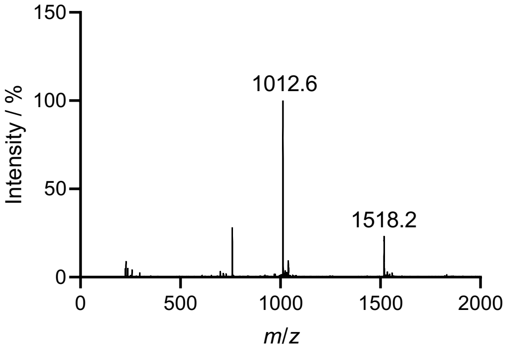

Left: HPLC chromatogram at λ = 210 nm, peak retention time: 12.2 min, gradient: 5–95 % ACN in 30 min; Right: MS spectrum, *MW*: 3035.80 g·mol^{-1}, calc. *m*/*z*: 1517.76 / 1012.17.

24γ

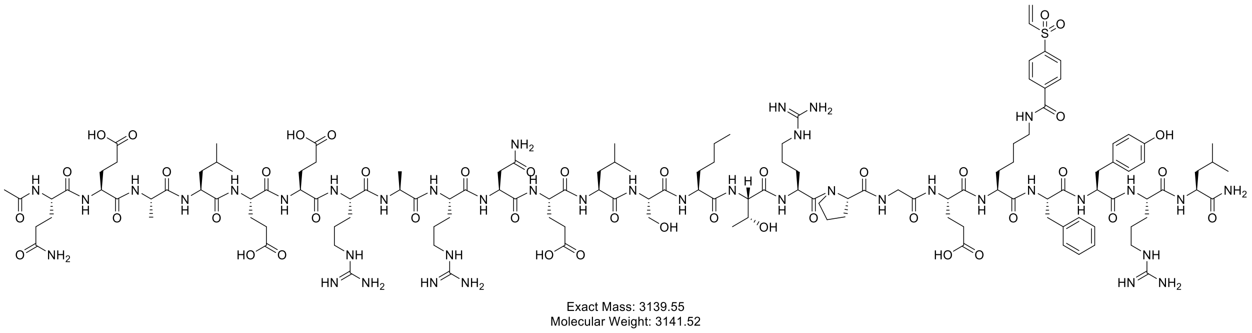

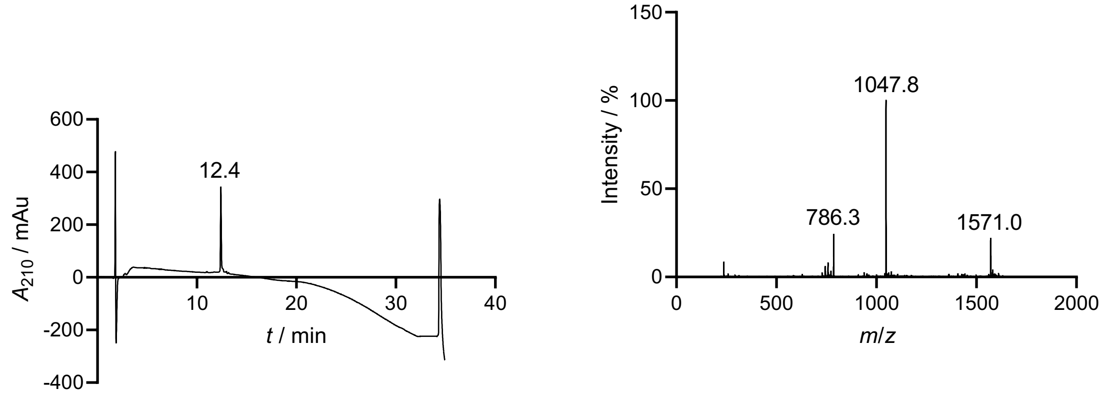

Left: HPLC chromatogram at λ = 210 nm, peak retention time: 12.4 min, gradient: 5–95 % ACN in 30 min; Right: MS spectrum, *MW*: 3131.53 g·mol^{-1}, calc. *m*/*z*: 1570.78 / 1047.52 / 785.89.

24δ

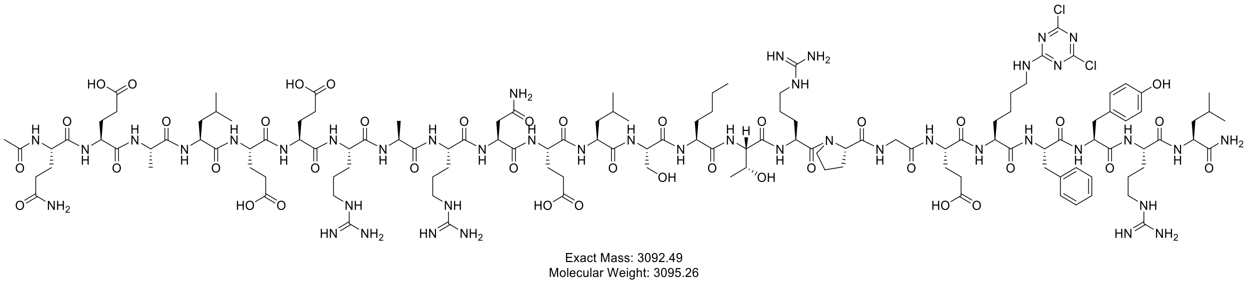

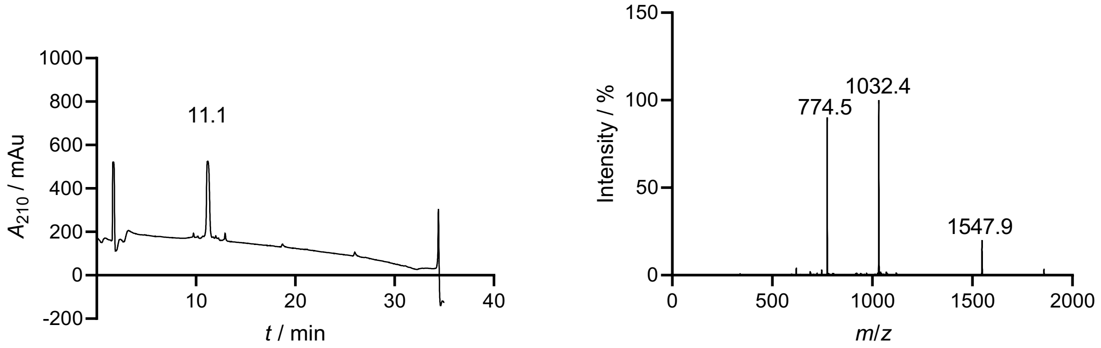

Left: HPLC chromatogram at λ = 210 nm, peak retention time: 11.1 min, gradient: 5–95 % ACN in 30 min; Right: MS spectrum, *MW*: 3095.26 g·mol^{-1}, calc. *m*/*z*: 1547.25 / 1031.83.

24fα

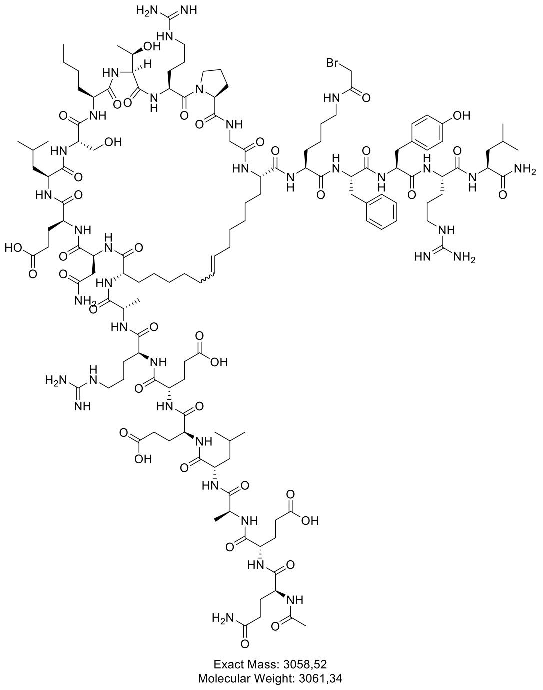

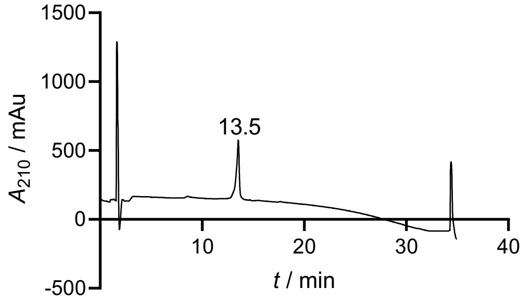

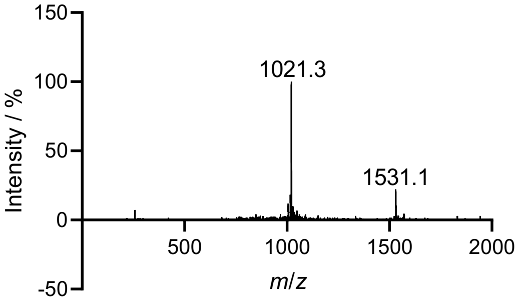

Left: HPLC chromatogram at λ = 210 nm, peak retention time: 13.5 min, gradient: 5–95 % ACN in 30 min; Right: MS spectrum, *MW*: 3058.52 g·mol^{-1}, calc. *m*/*z*: 1530.26 / 1020.51.

24fβ

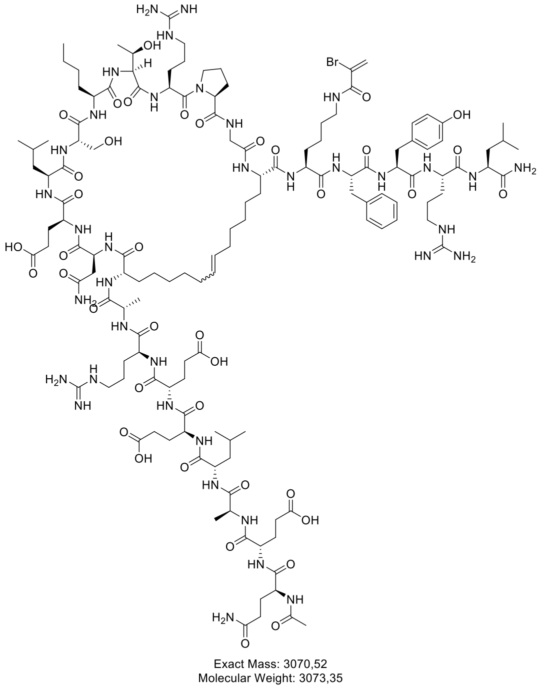

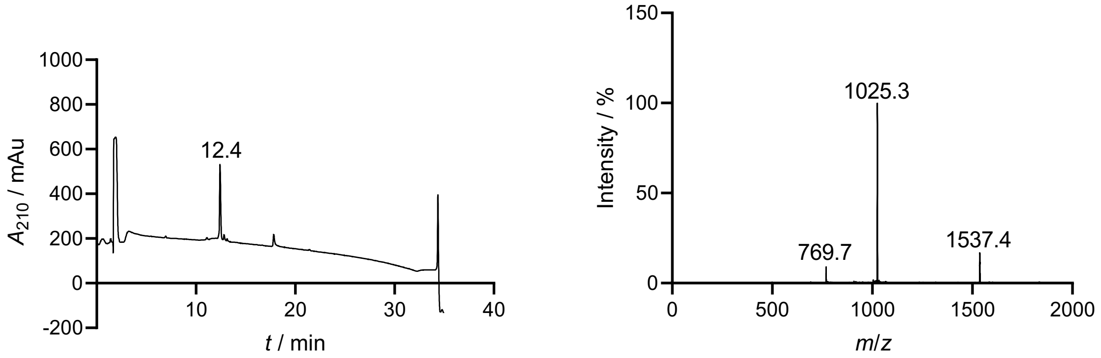

Left: HPLC chromatogram at λ = 210 nm, peak retention time: 12.4 min, gradient: 5–95 % ACN in 30 min; Right: MS spectrum, *MW*: 3073.35 g·mol^{-1}, calc. *m*/*z*: 1536.26 / 1024.51 / 768.6.

24fγ

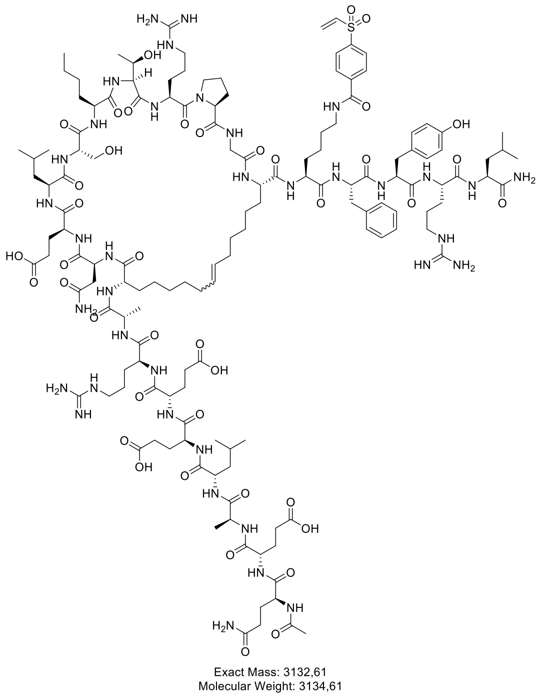

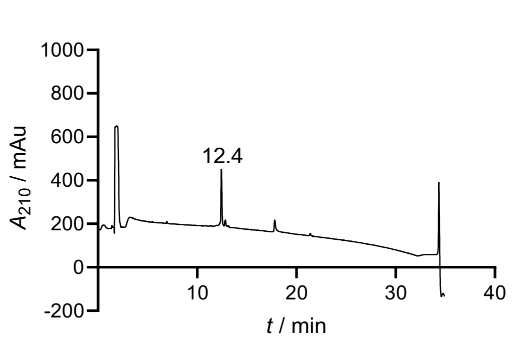

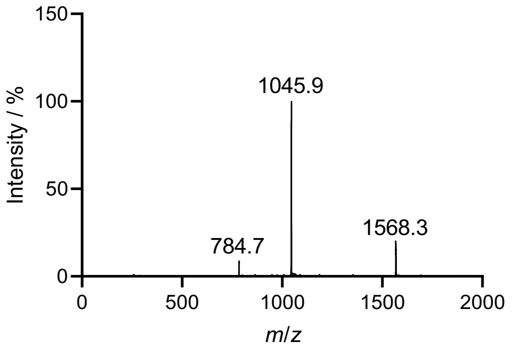

Left: HPLC chromatogram at λ = 210 nm, peak retention time: 12.4 min, gradient: 5–95 % ACN in 30 min; Right: MS spectrum, *MW*: 3134.61 g·mol^{-1}, calc. *m*/*z*: 1567.3 / 1045.2.

24fδ

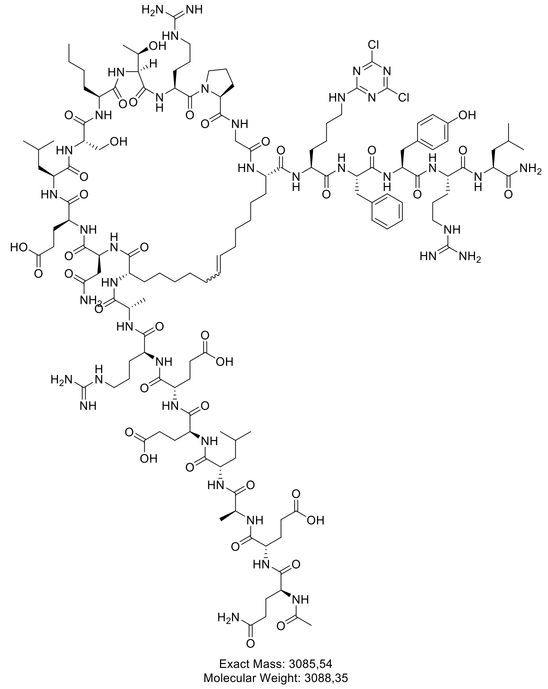

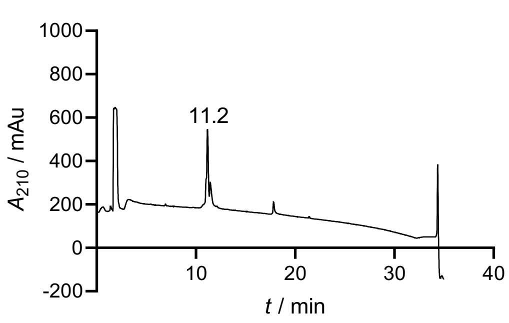

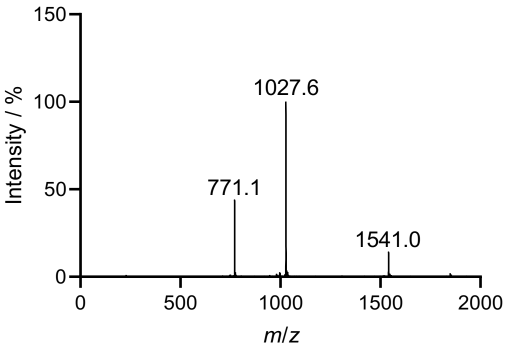

Left: HPLC chromatogram at λ = 210 nm, peak retention time: 11.2 min, gradient: 5–95 % ACN in 30 min; Right: MS spectrum, *MW*: 3088.35 g·mol^{-1}, calc. *m*/*z*: 1543.8 / 1029.5 / 772.4.

20fα

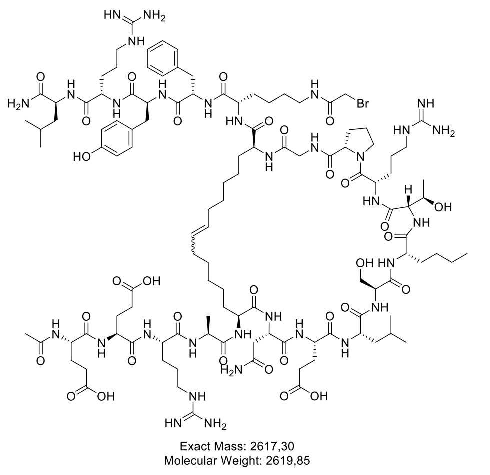

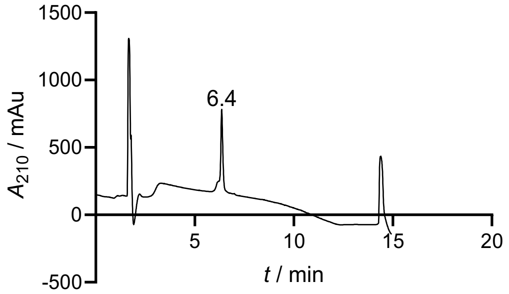

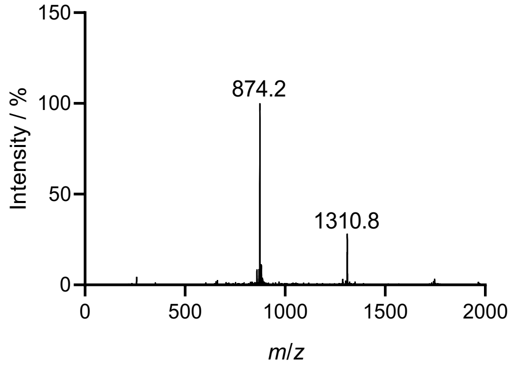

Left: HPLC chromatogram at λ = 210 nm, peak retention time: 6.4 min, gradient: 5–95 % ACN in 10 min; Right: MS spectrum, MW: 2619.85 g·mol-1, calc. m/z: 1309.65 / 873.43.

17fα

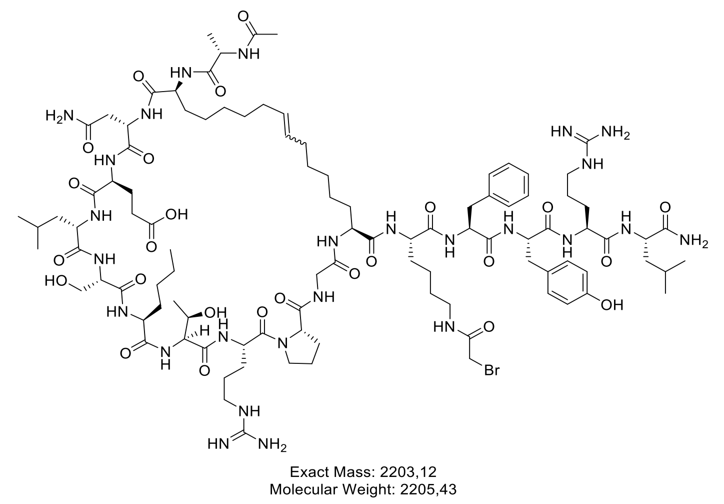

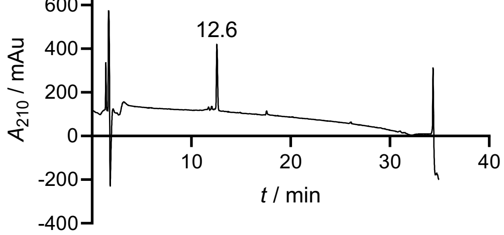

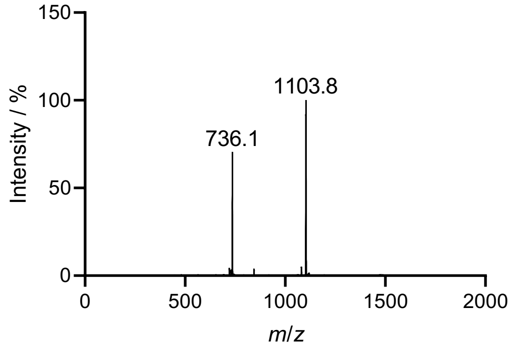

Left: HPLC chromatogram at λ = 210 nm, peak retention time: 12.6 min, gradient: 5–95 % ACN in 30 min; Right: MS spectrum, *MW*: 2205.43 g·mol^{-1}, calc. *m*/*z*: 1102.6 / 735.4.

17fβ

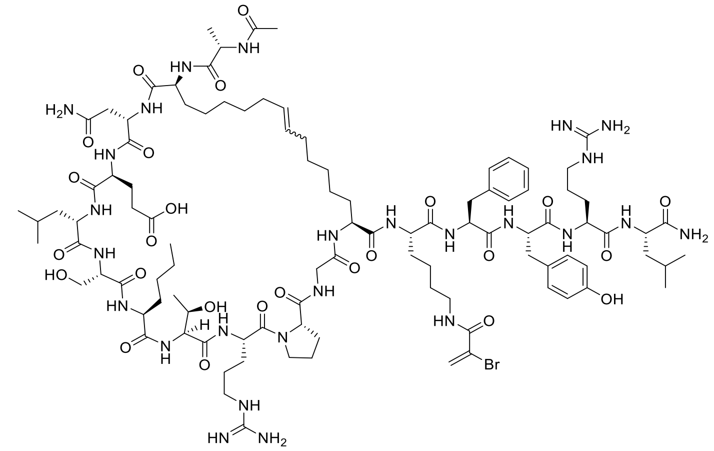

Exact Mass: 2215,12
Molecular Weight: 2217,44

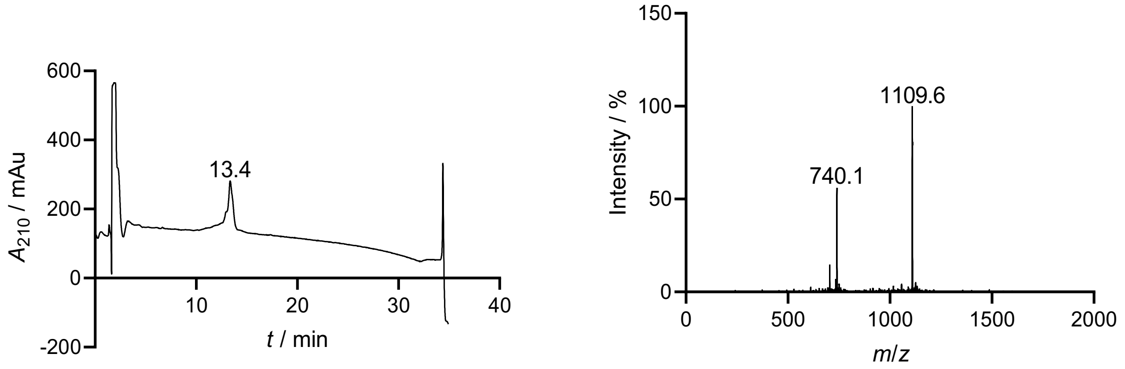

Left: HPLC chromatogram at λ = 210 nm, peak retention time: 13.4 min, gradient: 5–95 % ACN in 30 min; Right: MS spectrum, *MW*: 2217.44 g·mol^{-1}, calc. *m*/*z*: 1108.56 / 739.4.

17fγ

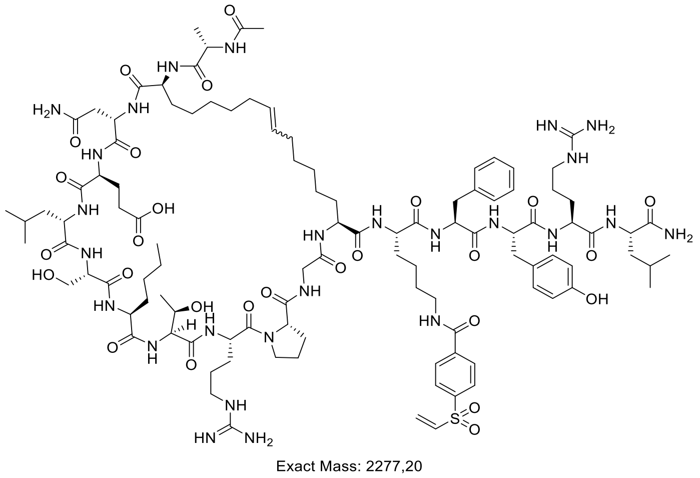

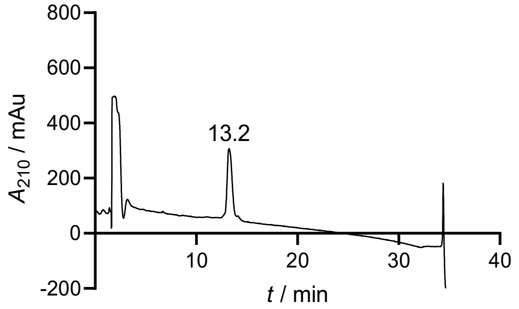

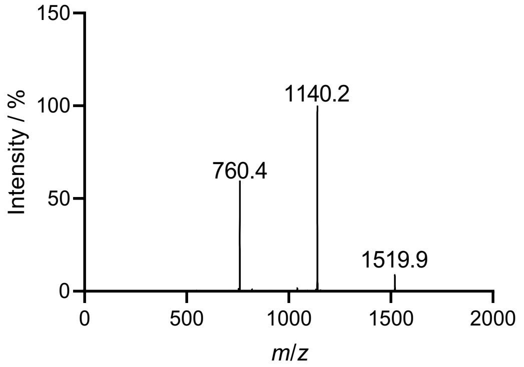

Left: HPLC chromatogram at λ = 210 nm, peak retention time: 13.2 min, gradient: 5–95 % ACN in 30 min; Right: MS spectrum, *MW*: 2278.20 g·mol^{-1}, calc. *m*/*z*: 1139.6 / 760.0.

17fδ

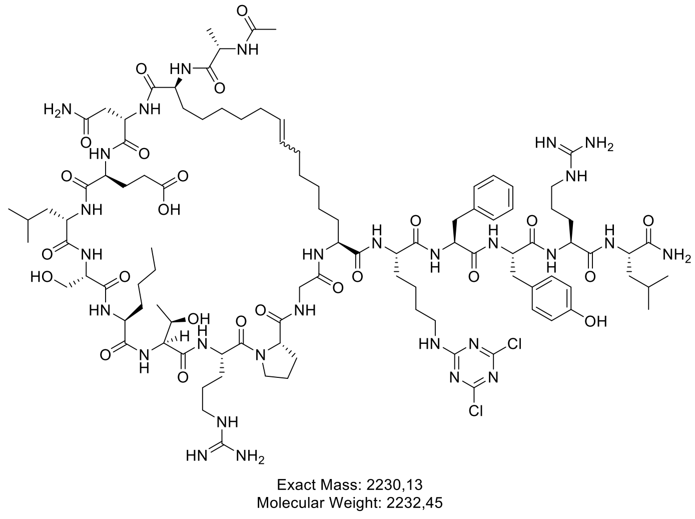

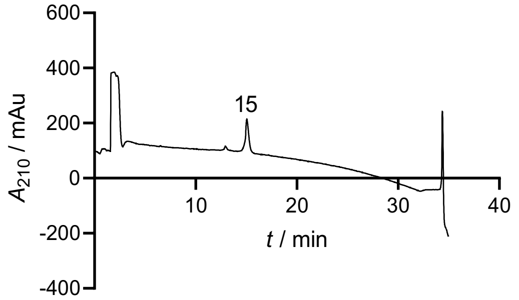

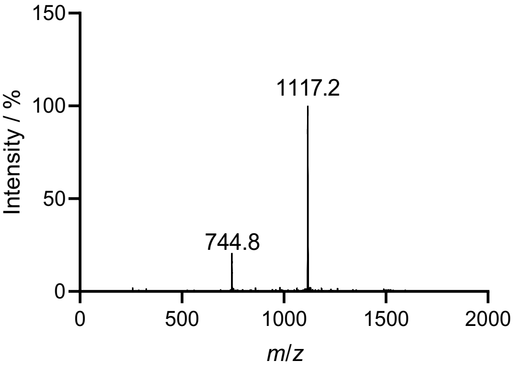

Left: HPLC chromatogram at λ = 210 nm, peak retention time: 15 min, gradient: 5–95 % ACN in 30 min; Right: MS spectrum, *MW*: 2232.45 g·mol^{-1}, calc. *m*/*z*: 1116.06 / 744.38.

15fα

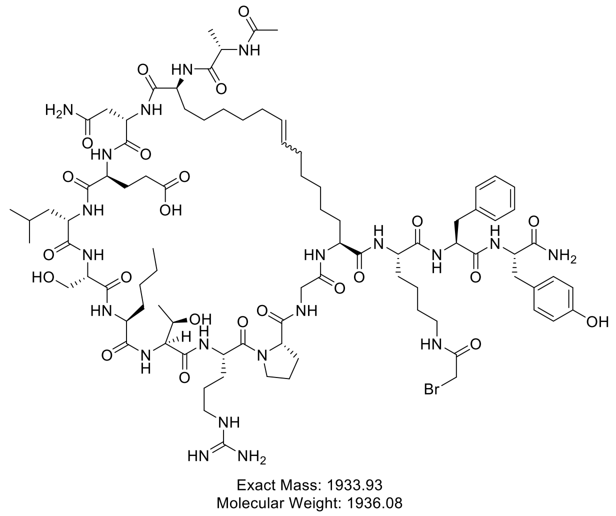

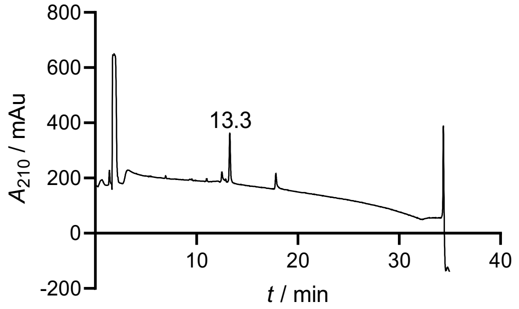

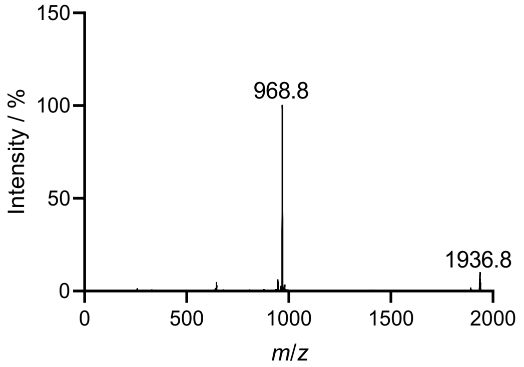

Left: HPLC chromatogram at λ = 210 nm, peak retention time: 13.3 min, gradient: 5–95 % ACN in 30 min; Right: MS spectrum, *MW*: 1936.08 g·mol^{-1}, calc. *m*/*z*: 1934.93 / 967.97.

L8S1

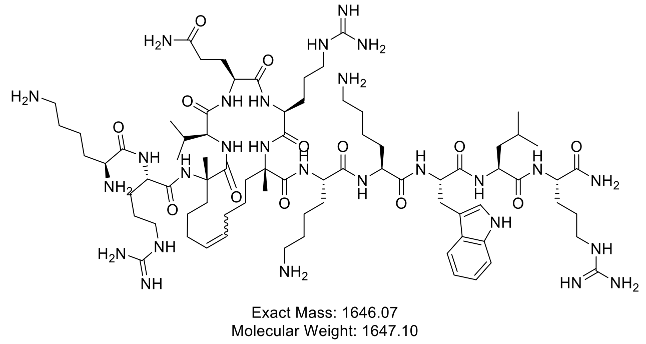

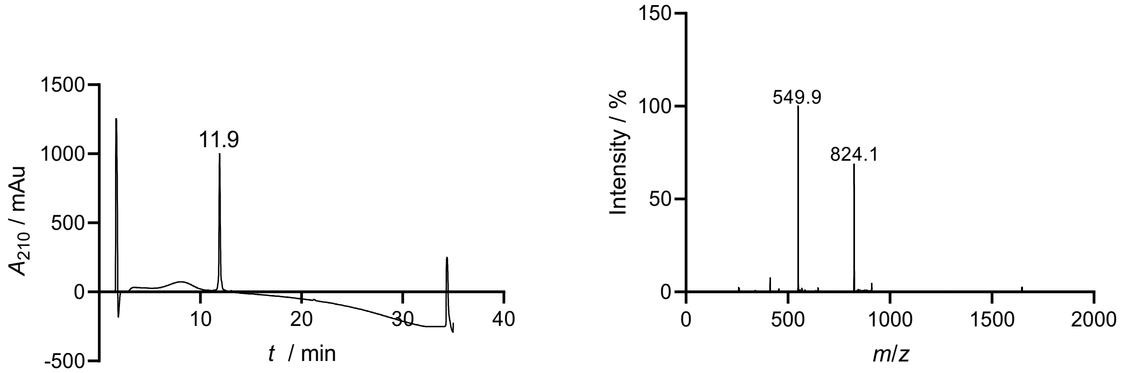

Left: HPLC chromatogram at λ = 210 nm, peak retention time: 11.9 min, gradient: 5–95 % ACN in 30 min; Right: MS spectrum, *MW*: 1647.10 g·mol^{-1}, calc. *m*/*z*: 824.1 / 549.7.

5. References

(1) Jeganathan, S.; Wendt, M.; Kiehstaller, S.; Brancaccio, D.; Kuepper, A.; Pospiech, N.; Carotenuto, A.; Novellino, E.; Hennig, S.; Grossmann, T. N. A Peptide with Fine-Tuned Flexibility Inhibits NF-Y Transcription Factor Assembly. *Angew. Chemie Int. Ed.* **2019**, *58*, 17351-17358. https://doi.org/10.1002/anie.201907901.

(2) Kureisaite-Ciziene, D.; Varadajan, A.; McLaughlin, S. H.; Glas, M.; Silva, A. M.; Luirink, R.; Mueller, C.; Blaauwen, T. Den; Grossmann, T. N.; Luirink, J.; Löwe, J.; den Blaauwen, T.; Grossmann, T. N.; Luirink, J.; Lowe, J. Structural Analysis of the Interaction between the Bacterial Cell Division Proteins FTSQ and FTSB. *MBio* **2018**, *9* (5), e01346-18. https://doi.org/10.1128/mBio.01346-18.

(3) Schouten, G. K.; Paulussen, F. M.; Kuipers, O. P.; Bitter, W.; Grossmann, T. N.; van Ulsen, P. Stapling of Peptides Potentiates: The Antibiotic Treatment of Acinetobacter Baumannii In Vivo. *Antibiotics* **2022**, *11* (2), 273. https://doi.org/10.3390/antibiotics11020273.

(4) Kim, Y. W.; Grossmann, T. N.; Verdine, G. L. Synthesis of All-Hydrocarbon Stapled ±-Helical Peptides by Ring-Closing Olefin Metathesis. *Nat. Protoc.* **2011**, *6* (6), 761–771. https://doi.org/10.1038/nprot.2011.324.

(5) Glas, M.; Bart Van Den Berg Van Saparoea, H.; McLaughlin, S. H.; Roseboom, W.; Liu, F.; Koningstein, G. M.; Fish, A.; Den Blaauwen, T.; Heck, A. J. R.; De Jong, L.; Bitter, W.; De Esch, I. J. P.; Luirink, J. The Soluble Periplasmic Domains of Escherichia Coli Cell Division Proteins FtsQ/FtsB/FtsL Form a Trimeric Complex with Submicromolar Affinity. *J. Biol. Chem.* **2015**, *290* (35), 21498–21509. https://doi.org/10.1074/jbc.M115.654756.

(6) Hanwell, M. D.; Curtis, D. E.; Lonie, D. C.; Vandermeersch, T.; Zurek, E.; Hutchison, G. R. *SOFTWARE Open Access Avogadro: An Advanced Semantic Chemical Editor, Visualization, and Analysis Platform*; 2012; Vol. 4.

(7) D.A. Case, K. Belfon, I.Y. Ben-Shalom, S.R. Brozell, D.S. Cerutti, T.E. Cheatham, III, V. W. D. C., T.A. Darden, R.E. Duke, G. Giambasu, M.K. Gilson, H. Gohlke, A.W. Goetz, R Harris, S. Izadi, S.A. Izmailov, K. Kasavajhala, A. Kovalenko, R. Krasny, T. Kur, P. A. K.; D.A. Case, K. Belfon, I.Y. Ben-Shalom, S.R. Brozell, D.S. Cerutti, T.E. Cheatham, III, V. W. D. C.; T.A. Darden, R.E. Duke, G. Giambasu, M.K. Gilson, H. Gohlke, A.W. Goetz,R Harris, S. Izadi, S. A. I.; K. Kasavajhala, A. Kovalenko, R. Krasny, T. Kurtzman, T.S. Lee, S. LeGrand, P. Li, C. Lin, J. L.; T. Luchko, R. Luo, V. Man, K.M. Merz, Y. Miao, O. Mikhailovskii, G. Monard, H. Nguyen, A. Onufriev, F.; Pan, S. Pantano, R. Qi, D.R. Roe, A. Roitberg, C. Sagui, S. Schott-Verdugo, J. Shen, C.L. Simmerling, N. R.; Skrynnikov, J. Smith, J. Swails, R.C. Walker, J. Wang, L. Wilson, R.M. Wolf, X. Wu, Y. Xiong, Y. X.; D.M. York and P.A. Kollman (**2020**), AMBER 2020, University of California, S. F. AMBER 2020. *Journal of Chemical Information and Modeling*. University of California: San Francisco, San Fransisco 2020.

(8) Khoury, G. A.; Smadbeck, J.; Tamamis, P.; Vandris, A. C.; Kieslich, C. A.; Floudas, C. A. Forcefield_NCAA: Ab Initio Charge to Aid in the Discovery and Design of Therapeutic Proteinsand

Peptides with Unnatural Amino Acids and Their Application to Complement of the Compstatin Family. *ACS Synth. Biol.* **2014**, *3* (12), 855. https://doi.org/10.1021/SB400168U.

(9) Anandakrishnan, R.; Aguilar, B.; Onufriev, A. V. H++ 3.0: Automating PK Prediction and the Preparation of Biomolecular Structures for Atomistic Molecular Modeling and Simulations. *Nucleic Acids Res.* **2012**, *40* (W1), W537–W541. https://doi.org/10.1093/nar/gks375.

(10) Buncherd, H.; Roseboom, W.; Ghavim, B.; Du, W.; de Koning, L. J.; de Koster, C. G.; de Jong, L. Isolation of Cross-Linked Peptides by Diagonal Strong Cation Exchange Chromatography for Protein Complex Topology Studies by Peptide Fragment Fingerprinting from Large Sequence Databases. *J. Chromatogr. A* **2014**, *1348*, 34–46. https://doi.org/10.1016/j.chroma.2014.04.083.

(11) Perkins, D. N.; Pappin, D. J. C.; Creasy, D. M.; Cottrell, J. S. Probability-based Protein Identification by Searching Sequence Databases Using Mass Spectrometry Data - Perkins - 1999 - ELECTROPHORESIS - Wiley Online Library. *Electrophoresis* **1999**, *20*, 3551–3567.

(12) Panchaud, A.; Singh, P.; Shaffer, S. A.; Goodlett, D. R. XComb: A Cross-Linked Peptide Database Approach to Protein-Protein Interaction Analysis. *J. Proteome Res.* **2010**, *9* (5), 2508–2515. https://doi.org/10.1021/pr9011816.

(13) Sampson, B. A.; Misra, R.; Benson, S. A. Identification and Characterization of a New Gene of Escherichia Coli K-12 Involved in Outer Membrane Permeability. *Genetics* **1989**, *122* (3), 491–501. https://doi.org/10.1093/GENETICS/122.3.491.

(14) Meiresonne, N. Y.; Consoli, E.; Mertens, L. M. Y.; Chertkova, A. O.; Goedhart, J.; den Blaauwen, T. Superfolder MTurquoise2 Ox Optimized for the Bacterial Periplasm Allows High Efficiency in Vivo FRET of Cell Division Antibiotic Targets. *Mol. Microbiol.* **2019**, *111* (4), 1025–1038. https://doi.org/10.1111/MMI.14206.

(15) Koppelman, C. M.; Aarsman, M. E. G.; Postmus, J.; Pas, E.; Muijsers, A. O.; Scheffers, D. J.; Nanninga, N.; Den Blaauwen, T. R174 of Escherichia Coli FtsZ Is Involved in Membrane Interaction and Protofilament Bundling, and Is Essential for Cell Division. *Mol. Microbiol.* **2004**, *51* (3), 645–657. https://doi.org/10.1046/J.1365-2958.2003.03876.X.

(16) Edelstein, A.; Amodaj, N.; Hoover, K.; Vale, R.; Stuurman, N. Computer Control of Microscopes Using MManager. *Curr. Protoc. Mol. Biol.* **2010**, *92* (1), 14.20.1-14.20.17. https://doi.org/10.1002/0471142727.MB1420S92.

(17) Vischer, N. O. E.; Verheul, J.; Postma, M.; van den Berg van Saparoea, B.; Galli, E.; Natale, P.; Gerdes, K.; Luirink, J.; Vollmer, W.; Vicente, M.; den Blaauwen, T. Cell Age Dependent Concentration of Escherichia Coli Divisome Proteins Analyzed with ImageJ and ObjectJ. *Front. Microbiol.* **2015**, *6* (JUN), 586. https://doi.org/10.3389/fmicb.2015.00586.

(18) Molecular Operating Environment (MOE). Chemical Computing Group ULC: 1010 Sherbooke St. West, Suite #910, Montreal, QC, Canada, H3A 2R7 2022.

(19) Wang, J.; Cieplak, P.; Kollman, P. A. How Well Does a Restrained Electrostatic Potential (RESP)

Model Perform in Calculating Conformational Energies of Organic and Biological Molecules? *J. Comput. Chem.* **2000**, *21* (12), 1049–1074. https://doi.org/10.1002/1096-987X(200009)21:12<1049::AID-JCC3>3.0.CO;2-F.

(20) Blosser, S. L.; Sawyer, N.; Maksimovic, I.; Ghosh, B.; Arora, P. S. Covalent and Noncovalent Targeting of the Tcf4/β-Catenin Strand Interface with β-Hairpin Mimics. *ACS Chem. Biol.* **2021**, *16* (8), 1518–1525. https://doi.org/10.1021/acschembio.1c00389.

(21) Lonsdale, R.; Burgess, J.; Colclough, N.; Davies, N. L.; Lenz, E. M.; Orton, A. L.; Ward, R. A. Expanding the Armory: Predicting and Tuning Covalent Warhead Reactivity. *J. Chem. Inf. Model.* **2017**, *57* (12), 3124–3137. https://doi.org/10.1021/acs.jcim.7b00553.

(22) Pettinger, J.; Jones, K.; Cheeseman, M. D. Lysine-Targeting Covalent Inhibitors. *Angew. Chemie - Int. Ed.* **2017**, *56* (48), 15200–15209. https://doi.org/10.1002/anie.201707630.

(23) Ray, S.; Murkin, A. S. New Electrophiles and Strategies for Mechanism-Based and Targeted Covalent Inhibitor Design. *Biochemistry* **2019**, *58* (52), 5234–5244. https://doi.org/10.1021/acs.biochem.9b00293.

(24) Flanagan, M. E.; Abramite, J. A.; Anderson, D. P.; Aulabaugh, A.; Dahal, U. P.; Gilbert, A. M.; Li, C.; Montgomery, J.; Oppenheimer, S. R.; Ryder, T.; Schuff, B. P.; Uccello, D. P.; Walker, G. S.; Wu, Y.; Brown, M. F.; Chen, J. M.; Hayward, M. M.; Noe, M. C.; Obach, R. S.; Philippe, L.; Shanmugasundaram, V.; Shapiro, M. J.; Starr, J.; Stroh, J.; Che, Y. Chemical and Computational Methods for the Characterization of Covalent Reactive Groups for the Prospective Design of Irreversible Inhibitors. *J. Med. Chem.* **2014**, *57* (23), 10072–10079. https://doi.org/10.1021/jm501412a.

(25) Cuesta, A.; Taunton, J. Lysine-Targeted Inhibitors and Chemoproteomic Probes. *Annu. Rev. Biochem.* **2019**, *88*, 365-381. https://doi.org/10.1146/annurev-biochem-061516-044805.

(26) Martin, J. S.; MacKenzie, C. J.; Fletcher, D.; Gilbert, I. H. Characterising Covalent Warhead Reactivity. *Bioorganic Med. Chem.* **2019**, *27* (10), 2066–2074. https://doi.org/10.1016/j.bmc.2019.04.002.

(27) Gambini, L.; Baggio, C.; Udompholkul, P.; Jossart, J.; Salem, A. F.; Perry, J. J. P.; Pellecchia, M. Covalent Inhibitors of Protein–Protein Interactions Targeting Lysine, Tyrosine, or Histidine Residues. *J. Med. Chem.* **2019**, *62* (11), 5616–5627. https://doi.org/10.1021/acs.jmedchem.9b00561.

(28) Martín-Gago, P.; Olsen, C. A. Arylfluorosulfate-Based Electrophiles for Covalent Protein Labeling: A New Addition to the Arsenal. *Angew. Chem. Int. Ed.* **2018**, *58*, 957–966. https://doi.org/10.1002/anie.201806037.

(29) Shannon, D. A.; Weerapana, E. Covalent Protein Modification: The Current Landscape of Residue-Specific Electrophiles. *Current Opinion in Chemical Biology*. Elsevier Ltd 2015, pp 18–26. https://doi.org/10.1016/j.cbpa.2014.10.021.

(30) Yoo, D. Y.; Hauser, A. D.; Joy, S. T.; Bar-Sagi, D.; Arora, P. S. Covalent Targeting of Ras G12C by Rationally Designed Peptidomimetics. *ACS Chem. Biol.* **2020**, *15* (6), 1604–1612. https://doi.org/10.1021/acschembio.0c00204.

(31) Gehringer, M.; Laufer, S. A. Emerging and Re-Emerging Warheads for Targeted Covalent Inhibitors: Applications in Medicinal Chemistry and Chemical Biology. *J. Med. Chem.* **2019**, *62* (12), 5673–5724. https://doi.org/10.1021/acs.jmedchem.8b01153.
